# Wearable Fluidic Fabric with Excellent Heat Transfer Performance for Sports Recovery

**DOI:** 10.1002/advs.202411691

**Published:** 2025-01-07

**Authors:** Jing Yang, Ying Xiong, Jinli Piao, Manyui Leung, Guosai Liu, Mingyue Zhu, Shengyang Tang, Lisha Zhang, Xiaoming Tao

**Affiliations:** ^1^ Research Institute for Intelligent Wearable Systems School of Fashion and Textiles The Hong Kong Polytechnic University Kowloon 999077 China

**Keywords:** heat transfer model, heat transfer rate and effectiveness, skin cooling and heating, textile‐based flexible heat transfer panel, wearable fluidic fabric

## Abstract

Rapid temperature contrast hydrotherapy by water immersion has been utilized by athletes for effective sports recovery. However, its application at some training or competition venues is limited by high water consumption, bucky size, personal hygiene, and inconvenience. Here, a novel portable system equipped with highly effective, lightweight, and hygienic wearable fluidic fabric device is reported, that replaces direct water immersion. The measured heat transfer coefficient between the skin and the fabric is 98.5 W m^−2^ K^−1^, which is 92% of that in direct water immersion at 10 °C and significantly higher than that by previously reported cooling garments. The core layer, a flexible heat transfer panel (FHTP), can switch between cold and hot modes (5–40 °C) over an area of 0.3 m^2^. The contact condition between skin and the deformable FHTP has been considered in a new verified heat transfer model. Optimization of the parameters has resulted in excellent heat transfer performance. This fluidic fabric also holds potential in diverse applications, such as enhancing thermal safety and comfort in extreme environments (e.g., personal thermal management systems and fire‐protection suits), supporting cryotherapy and thermotherapy in rehabilitation and healthcare, and simulation of total tactile sensations in virtual reality.

## Graphical abstract

A novel wearable system with a lightweight, hygienic fluidic fabric effectively replaces direct water immersion for sports recovery. Comprising an inner ultra‐thin fabric, core FHTP, and outer thermal insulation fabric, the fluidic fabric efficiently cools and heats large areas like the thigh. It also suits thermal management, healthcare, and virtual reality applications.

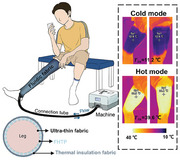

## 1 Introduction

**Figure 1:**
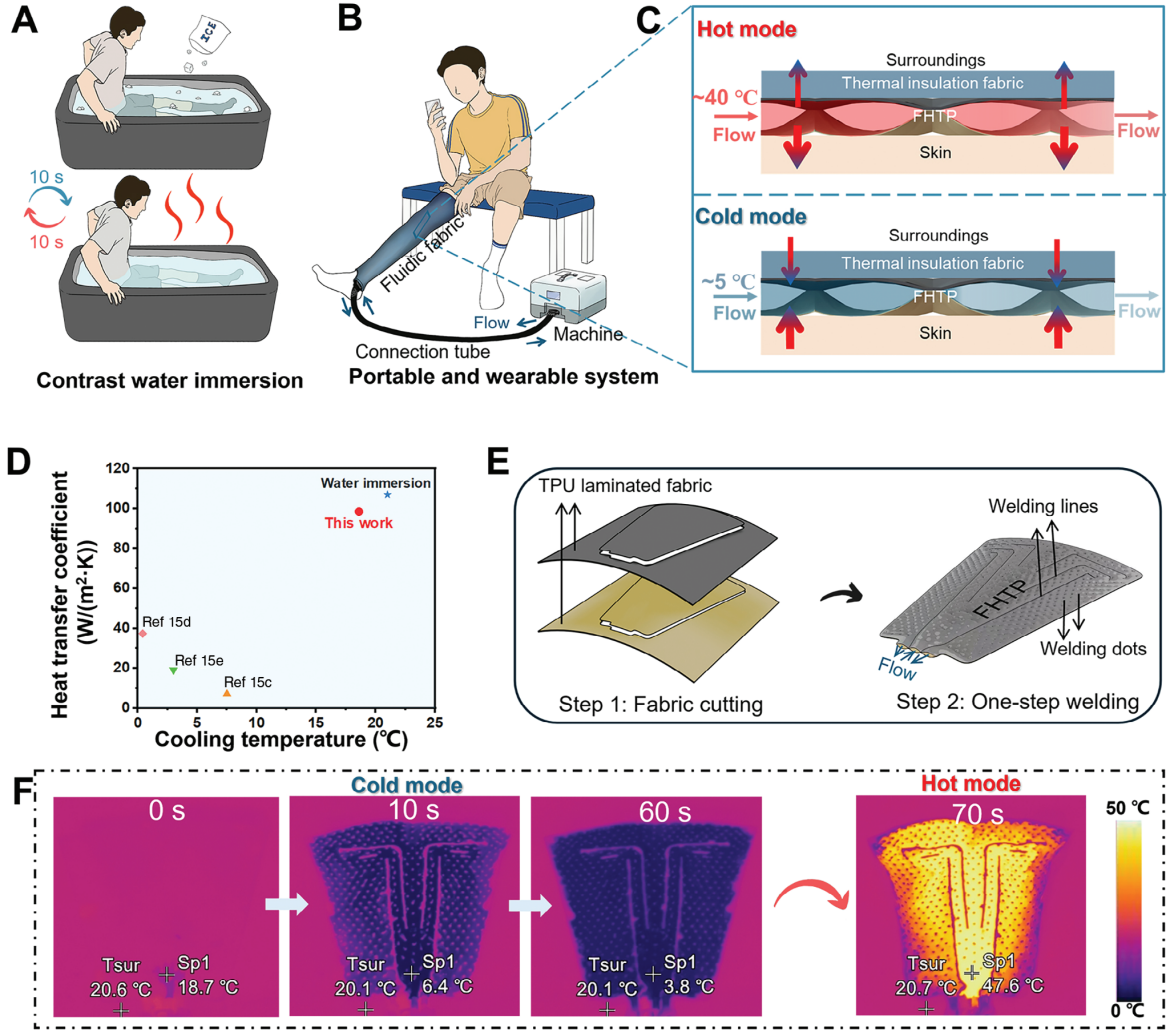
Schematics of COOLWEAR system, contrast water immersion and fabrication of a FHTP, as well as IR images of fast switch between cold and hot modes of a FHTP. A) Schematic of traditional water immersion of lower limbs for rapid contrast therapy. The water‐immersed therapy requires two buckets and a total of ≈100 L water. B) Schematic of the proposed portable and wearable system for rapid contrast therapy. This system comprises wearable fluidic fabrics, a machine, and a connection tube. Water is pumped from the tank in the control machine into the fluidic fabrics, and then recirculated back to the tank. C) Schematics of the cross‐section of wearable fluidic fabrics in cold and hot modes. The wearable fluidic fabric consists of a FHTP and a piece of thermal insulation fabric. When cold or warm water flows through the fluid channel of the FHTP, heat is transferred from high to low‐temperature regions, thereby cooling or warming the skin and the surroundings. The heat flux is indicated by arrows. D) Comparison of heat transfer performance, including the heat transfer coefficient between skin and fluidic fabric, as well as the cooling temperature of the skin, among our fluidic fabric, traditional water immersion, and other fluid cooling garments. Cooling temperature refers to the difference in skin temperature before and after the cooling mode. E) Two steps of the large‐scale production process of a FHTP: cutting two pieces of laminated fabrics; then, one‐step welding of the fabrics along the designed lines and at the dots. F) IR images of a FHTP have been captured during a cooling/heating cycle, when the FHTP was connected to the machine running a rapid contrast therapy program. The measurement began when water was pumped into the FHTP. Sp1 denotes the temperature at the point where water flows in. *T_sur_* represents the temperature of the surroundings.

Contrast water therapy, that is alternation between hot‐ and cold‐water immersion, has been a common method to facilitate recovery of athletes after physical training or competition.^[^ ^1^ ^]^ It can reduce fatigue^[^ ^2^ ^]^ and swelling,^[^ ^3^ ^]^ alleviate symptoms of exercise‐induced muscle soreness.^[^ ^1^, ^4^ ^]^ As shown in **Figure** **1**A, contrast therapy requires two buckets full of cold water of 10–15 °C and hot water of 38–40 °C, respectively. The recommended alternation time between cold and hot water immersion is within 10 s. The total immersion duration is often 14–15 min.^[^ ^5^ ^]^ The total water volume for immersing lower limbs is 100 L.^[^ ^6^ ^]^ However, the application of such kind of hydrotherapy is restricted, because of substantial water consumption, poor portability, and potential hygiene problems caused by multi‐users. Therefore, one urgent demand is to develop a portable and wearable system that exhibits a similar therapy effect but overcomes the drawbacks of contrast water therapy. This system should fulfill the following requirements: saving water, portability, accessibility, hygiene, lightweight, large area for skin cooling/heating, rapid cooling, heating and transition, as well as high skin cooling/heating performance close to water‐immersed therapy.

Current wearable cooling/heating techniques for skin cooling or heating, including fluid (liquid/air) cooling garment,^[^ ^7^ ^]^ wearable thermoelectric (TE) devices,^[^ ^8^ ^]^ joule heating,^[^ ^9^ ^]^ and phase change materials,^[^ ^10^ ^]^ have yet to meet the above requirements simultaneously. Liquid cooling system has higher cooling rate than air cooling system and a more desirable shape than ice packs or phase change materials.^[^ ^11^ ^]^ However, the tube length over 90 m in a liquid cooling garment (LCG) makes it difficult to achieve rapid cooling or heating effect nor contrast therapy.^[^ ^12^ ^]^ Commercial TE device only achieves rapid switch between cooling and heating modes, when it is equipped with heavy heat dissipation units (Figure S1, Supporting Information). Nevertheless, the wearable TE devices can hardly provide large‐area cooling or heating effect.^[^ ^13^ ^]^ In addition, compared with hydrotherapy, these techniques show a much lower heat transfer rate between devices and human body. The heat transfer coefficient of water immersion was reported to be ≈107 W m^−2^ K^−1^;^[^ ^14^ ^]^ while it was estimated to range from 13 to 37 W m^−2^ K^−1^ for the reported LCGs.^[^ ^15^ ^]^ This estimation is based on dividing the heat transfer rate of the garment by the temperature difference between skin and working fluid. Details on the setup of LCGs and the estimation of the coefficient can be found in Table S1 (Supporting Information). Furthermore, such fluidic wearables lack an assessment standard. The common assessment criterion focuses on the total heat removed by fluid, which overestimates the heat transfer, since the fluid can also absorb/dissipate heat from/to surroundings with a lack of adequate insulation.

Heat transfer models have been extensively utilized in the optimization of wearable cooling and heating units.^[^ ^16^ ^]^ A numerical human thermoregulatory model integrated with thermal and comfort models was established and used to comprehensively study the design factors of liquid cooling garment in the hot environment.^[^ ^17^ ^]^ In addition, an analytical heat transfer model was built for identifying the main limitations on the performance of liquid cooling garment.^[^ ^15c^ ^]^ The interfacial contact conditions between skin and wearable devices are crucial for heat transfer performance; however, contact thermal resistance is often neglected in many studies.^[^ ^15^, ^16^, ^17^ ^]^

To address the aforementioned issues, an innovative textile‐based flexible heat transfer panel (FHTP) with network fluidic channels for rapid contrast therapy is designed and developed in this research. The FHTP for lower limbs is accessible, portable, lightweight (350 g), water saving (total water volume of 3 L for one limb). Using two FHTPs for two lower limbs consume only 6 L of water versus ≈100 L for immersion cooling. Moreover, it shows a rapid cooling/heating transition within 10 s over a large effective area of 0.3 m^2^. Additionally, a mathematical heat transfer model, incorporating contact pressure and area ratio raising from the FHTP deformation under various flow conditions, has been developed and studied to provide detailed insights into the heat transfer processes involving the body skin, the FHTP, and surroundings. This experimentally validated model helps identify the dominant factors on heat transfer performance, thereby providing guidelines for the optimization of the FHTP. To precisely describe the performance of the FHTP, two evaluation criteria are proposed: the heat transfer rate between the skin and the FHTP, and the FHTP's effectiveness. The first criterion focuses on the amount of heat removed or absorbed by the skin, rather than the total heat absorbed or dissipated by the fluid. The second criterion assesses the percentage of heat from the fluid that is absorbed or dissipated into the skin, rather than into the surroundings. Furthermore, one wearable fluidic fabric that is fabricated by combining a FHTP and a wrap together demonstrates superior heat transfer performance, when it is used on a participant's thigh. Its heat transfer coefficient is ≈98.5 W m^−2^ K^−1^, which is comparable to that of cold‐water immersion (≈107 W m^−2^ K^−1^).

## 2 Results and Discussion

### 2.1 Structure and Performance of the FHTP

The cold or warm water from the small tank in the portable machine circulates in the FHTP inside the wearable fluidic fabric and flows back to the tank via connection tube (Figure 1B). The heat transfer between skin and the FHTP occurs mainly via heat conduction. The flowing water inside the FHTP can absorb heat from skin and surroundings on cold mode and dissipate heat into skin and surroundings on hot mode. The upper surface of the FHTP is covered by a piece of thermal insulation fabric to reduce undesirable heat transfer between the FHTP and surroundings (Figure 1C). This wearable fluidic fabric demonstrates a significantly higher skin heat transfer coefficient and cooling temperature compared to other LCGs.^[^ ^15^ ^]^ With an average heat transfer coefficient of 98.5 W m^−2^ K^−1^ (details on the measurement listed in Table S1, Supporting Information), it achieves 92% of the efficiency reported for cold water immersion (≈107 W m^−2^ K^−1^).^[^ ^14a^ ^]^ Additionally, the skin cooling temperature of our fluidic fabric is comparable to that achieved in cold water immersion (Figure 1D). The design of appropriate fluid channel pattern is crucial to the FHTP achieving uniform and rapid cooling/heating effect. Thus, serpentine and network patterns were designed and fabricated (Figure S2, Supporting Information). The FHTP in network pattern proves to have more uniform temperature distribution and faster flow rate than the one in serpentine pattern (Table S2, Supporting Information). The easy and large scalable fabrication process of the FHTP only involves two steps: fabric cutting and welding (Figure 1E). Two pieces of thermoplastic polyurethane (TPU) laminated fabrics are cut with their TPU surfaces facing each other. Then, the fabrics are one‐step welded together along lines and at dots to construct FHTP. Welding lines serve as guide vanes to direct the main flow direction. Welding dots play an important role in limiting the over‐expansion of flexible fluid channels and reducing the water volume in the channels. As a result, the water volume in the FHTP for a lower limb is as low as 1.2 L in an effective area of 0.3 m^2^ (Figure S3, Supporting Information). For rapid contrast therapy, each cooling/heating cycle includes two modes: a cold mode with temperature of ≈5 °C for 1 min, and a hot mode with temperature of ≈40 °C for 2 min. The surface temperature change of the FHTP in five cycles was recorded by an IR camera (Supplementary Video S1), which indicates the rapid cooling and heating transition. The water can flow out of the FHTP within 10 s in two modes (Figure 1F). The surface temperature of the FHTP is evenly distributed within 15 s, which maintains until mode transition (Figure S4, Supporting Information). The rapid change and even distribution of temperature can fulfill the therapy requirements of sports recovery. Moreover, the FHTP offers desirable accessibility, allowing users to switch between cold and hot modes without needing to move their lower limbs from one bucket to another, unlike traditional contrast water therapy.

### 2.2 Heat Transfer Model of the FHTP and Experimental Validation

**Figure 2:**
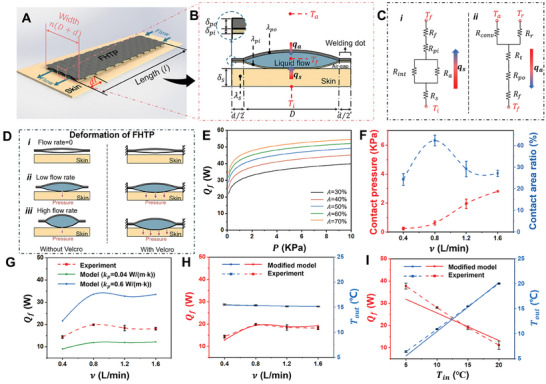
The FHTP unit, equivalent thermal resistance network diagram, as well as analytical and experimental results. A) Schematic of a rectangular area in a FHTP. The distance between flow inlet and outlet in this area is defined as the channel length (*l*). The width of the rectangular is *n(D+d)*. B) Schematic of the quasi‐elliptical cross‐section of the FHTP unit. The heat flux from skin and surroundings to the cold liquid flow (*q_s_* and *q_a_*) is indicated by arrows. C) Equivalent thermal resistance network diagram of the FHTP unit in B). (*i*) depicts the thermal resistance network from the bottom of skin to the fluid in the FHTP channel. (*ii*) depicts the thermal resistance network from the fluid in the FHTP channel to the surroundings. In the heat transfer model, *T_a_* is equal to *T_r_*. D) Illustration of the FHTP deformation at different inlet water flow rates, both with and without Velcro. As water is pumped into the flexible fluid channel, it expands and applies pressure to the underlying skin, assisted by Velcro. The pressure applied to the skin and the contact area between skin and the FHTP vary under different flow rates. E) Analytical results of heat transfer rate of fluid (*Q_f_*) as a function of the contact pressure under different contact area ratios. F) The experimental results of the influence of water flow rates (*v*) on contact pressure and contact area ratio at the interface between skin and the FHTP. G) Experimental and analytical results of heat transfer rate of fluid (*Q_f_*) under different flow rates. The influence of flow rates on the contact pressure and area ratio is incorporated in the heat transfer model. The analytical results for *Q_f_* are obtained by considering the thermal conductivity of the FHTP as the thermal conductivity of the inner fabric (0.04 W m^−1^ K^−1^) and the flowing water (0.6 W m^−1^ K^−1^), respectively. H) Experimental and modified analytical results of *Q_f_* and *T_out_* under different flow rates. A correction factor (*f_int_*) with a value of 0.375 is introduced to the interfacial thermal conductivity between the FHTP and skin in the model to align the analytical results with the experimental results. I) Experimental and analytical results of the influence of inlet water temperature (*T_in_*) on *Q_f_* and *T_out_* (*v* = 0.8 L min^−1^).

For enhancing understanding of heat transfer between such novel FHTP and skin or surroundings, mathematical models are built and validated in this section. There are two basic elements, welding lines, and dots, in the network pattern of the FHTP (Figure S5A, Supporting Information). The area between two adjacent welding lines is chosen as the research object to establish the heat transfer model (Figure S5B, Supporting Information). **Figure** **2**A is the 3D schematic diagram of the research object when water is pumped into the channel. The width of the fluid channel is *n(D+d)*. The distance between water inlet and outlet is the channel length (*l*). The heat transfer rate of fluid (*Q_f_*) and outlet fluid temperature (*T_out_*) have been often used to validate the model prediction, which can be easily obtained by measuring the fluid temperature and flow rate in experiment.^[^ ^15b,d^ ^]^ The mathematical models on *Q_f_* and *T_out_* of the FHTP are built as the following (the detailed derivation can be found in Note S1, Supporting Information):

(1) $$\begin{array}{c} Q_{f}=c\rho \nu \cdot \frac{\alpha_{s}\cdot \left(T_{i}-T_{in}\right)+\alpha_{a}\cdot \left(T_{a}-T_{in}\right)}{\alpha_{s}+\alpha_{a}}\cdot \left(1-e^{-\frac{\left(\alpha_{s}+\alpha_{a}\right)\cdot n\left(D+d\right)}{c\rho \nu }\cdot l}\right) \end{array}$$

(2) $$\begin{array}{c} T_{out}=\left(T_{in}-\;\frac{\alpha_{s}\cdot T_{i}+\alpha_{a}\cdot T_{a}}{\alpha_{s}+\alpha_{a}}\right)\;\cdot e^{-\frac{\left(\alpha_{s}+\alpha_{a}\right)\cdot n\left(D+d\right)}{c\rho \nu }\cdot l}+\frac{\alpha_{s}\cdot T_{i}+\alpha_{a}\cdot T_{a}}{\alpha_{s}+\alpha_{a}} \end{array}$$

In Equations (1) and (2), the parameters, *α_s_* and *α_a_*, are analyzed by studying the unit (Figure 2B), which indicating the complex heat transfer between the FHTP and skin or surroundings in practice. The cross‐sectional shape of inflated channel is quasi‐elliptic (Figure S6, Supporting Information). When water is flowing into the fluid channel, air gaps exist between skin and welding dots of the FHTP. Heat is transferred mainly by conduction in the air gaps with the thickness of ≈4 mm.^[^ ^18^ ^]^ The thermal contact resistance between skin and inner surface of the FHTP (*R_int_*) is calculated based on the empirical equation (Equation S16, Supporting Information).^[^ ^19^ ^]^ The heat transfer pathway between dots and skin is ignored (Note S2, Figures S7 and S8, Supporting Information), because of two reasons: 1) the total area of dots is 5.7% of the whole area of the research object (0.18 × 0.24 m^2^); (2) the analytical heat transfer rate between dots and skin is less than 5%. The temperature under skin of 3 mm is regarded as a constant (*T_i_* = 35 °C), representing the core temperature. Between skin and fluid, the equivalent thermal resistance network includes the conductive thermal resistance of skin (*R_s_*), air gap (*R_a_*) and inner fabric of the FHTP (*R_pi_*), the interfacial contact thermal resistance between skin and the FHTP (*R_int_*), as well as the thermal resistance of forced convective heat transfer of water in the fluidic channel (*R_f_*) (Figure 2C(i)). Between the fluid and surroundings, there are the *R_f_*, the conductive thermal resistance of outer fabric (*R_po_*), and the thermal insulation fabric (*R_t_*), the thermal resistance of radiation (*R_r_*) and natural convection (*R_conv_*) (Figure 2C(ii)). *α_s_* and *α_a_* are derived as Equations (3) and (4) from the thermal resistance network (the detailed derivation can be found in Note S3, Supporting Information):

(3) $$\alpha_{s}=\frac{1}{R_{s}+\frac{R_{int}\cdot R_{a}}{A\cdot R_{a}+\left(1-A\right)\cdot R_{int}}+R_{pi}+R_{f}}\;$$

(4) $$\alpha_{a}=\frac{1}{R_{f}+R_{po}+R_{t}+\frac{R_{r}\cdot R_{conv}}{R_{r}+R_{conv}}}\;$$

The value of *Q_f_* and *T_out_* obtained via model and experiments under different water flow rates is compared, where *T_in_* and *T_a_* are kept at 15 °C and 22 ± 0.5 °C, respectively. An experimental setup, including the thermostatically controlled water circulation systems for curved platform and the rectangle FHTP, was built for measuring *Q_f_* and *T_out_* (Figures S9–S11, Supporting Information). In the experiment, *Q_f_* rises greatly before dropping slightly, when flow rate increases from 0.4 to 1.6 L min^−1^ (Figure 2G,H). The margin of error (*Er*) of the measured *Q_f_*, *Q_s_* and *Q_a_* is less than 10% under different flow rates (Table S3, Supporting Information), indicating the reliability of the measurement. Under various flow rates, *Re*, *Nu*, and *R_f_* predicted by modeling is plotted in Figure S12A–C (Supporting Information) (see Equation S17–S23 in Note S3, Supporting Information). The trend of *Q_f_* obtained from model and experiments is inconsistent (Figure S12D, Supporting Information), because the influence of flow rate is only focused on the *R_f_* in the model. Traditional heat transfer models have been established for a liquid cooling garment in a tube channel. However, in this work, the textile‐based channel is flexible. Its deformation is mainly caused by internal hydraulic pressure and external tensile force, as illustrated in Figure 2D. Without Velcro, the pressure applied by the FHTP on the skin is due to gravity alone. With Velcro, the internal hydraulic pressure also acts on the skin. As the flow rate increases, the internal hydraulic pressure rises. The deformation behavior of the FHTP may also influence the contact area between the FHTP and skin. The effect of contact pressure and area ratio are predicted by the heat transfer model. As contact pressure increases, *R_int_* initially drops dramatically and then levels off (Figure S13, Supporting Information). Consequently, *Q_f_* initially rises significantly and then exhibits a more gradual increase, assuming the area ratio remains constant (Figure 2E). Meanwhile, *Q_f_* also improves with an increase in the area ratio, when the contact pressure is held constant. The modeling results reveal that contact pressure and area ratio significantly affect *Q_f_*.

To measure the contact area ratio under different water flow rates, the dyed sodium alginate solution was painted on the inner fabric of the FHTP and the area that solution absorbed by A4 paper represents the contact area (Figure S14, Supporting Information). The effect of flow rate on the contact pressure and area ratio between skin and the FHTP is measured experimentally (Figure 2F and Table S4, Supporting Information). The contact pressure gradually increases with the increment of flow rate; whereas the largest contact area occurs when flow rate is 0.8 L min^−1^. When considering the effects of flow rate on the contact pressure, area ratio, and *R_f_* simultaneously, the trends in both the model and experimental results for *Q_f_* and *T_out_* are consistent (Figure 2G; Figure S15, Supporting Information). Two sets of model results are derived using different thermal conductivity values for the FHTP in the calculation of *R_int_*. The *R_int_* is calculated based on the empirical formula for finger‐object contact^[^ ^19^ ^]^ (Equation S16, Supporting Information). Different from finger‐contacting homogenous objects like metal and wood, the FHTP is inhomogeneous, which is composed of fabrics and flowing water. The experimental *Q_f_* value falls within the range of analytical *Q_f_* value when the thermal conductivity of the FHTP, assumed to be a homogeneous material, is set to 0.04 and 0.6 W m^−1^ K^−1^, corresponding to the conductivities of inner fabric and flowing water, respectively. To align the analytical results with the experimental findings, a correction factor (*f_int_*) is introduced to the interfacial thermal conductivity between the skin and the FHTP in the empirical formula for *R_int_* (Equation S16, Supporting Information). The experimental and analytical results for *Q_f_* and *T_out_* have been compared when *f_int_* ranged from 0.3 to 1, and the two results show high consistency when *f_int_* is set to 0.375 (Figure 2H) (The details can be found in Note S4 and Figure S16, Supporting Information). The modified heat transfer model is further validated by comparing with experimental results of *Q_f_* and *T_out_* under different inlet water temperature (*T_in_*), while the flow rate is kept at 0.8 L min^−1^. As shown in Figure 2I, the modified model is highly reliable, as *Q_f_* and *T_out_* from the model fit those from the experiment (Note S5 and Figure S17, Supporting Information). In the next section, the reliable model is used to quantitatively identify the primary factors on the heat transfer performance between the FHTP and skin.

### 2.3 Influencing Factors on the Heat Transfer Performance

**Figure 3:**
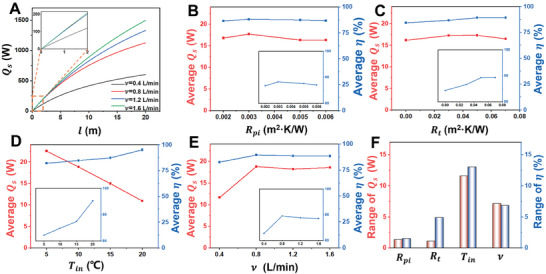
Model analysis of the heat transfer performance of the FHTP. A) Effect of the channel length on the heat transfer rate between skin and the FHTP (*Q_s_*) under flow rates of 0.4, 0.8, 1.2, and 1.6 L min^−1^. The inset is the magnified curves (*l*≤ 2 m). B) Effect of thermal resistance of inner fabric (*R_pi_*) on the average *Q_s_* and the average effectiveness of the FHTP (*η*). C) Effect of thermal resistance of thermal insulation fabric (*R_t_*) on the average *Q_s_* and *η*. D) Effect of inlet water temperature (*T_in_*) on the average *Q_s_* and *η*. E) Effect of inlet water flow rate (*v*) on the average *Q_s_* and *η*. The insets in (B–E) show the magnified average *η*. F) Importance of the influence of *R_pi_*, *R_t_*, *T_in_*, and *v* is compared by calculating the range of *Q_s_* and *η*, which is the difference between the maximum and minimum of the average *Q_s_* and *η*, respectively, in (B–E).

*Q_f_* often overestimates the performance of fluid cooling garment, because it includes the heat transfer between the fluid and surroundings. Thus, the heat transfer rate between skin and the FHTP (*Q_s_*), a more accurate mathematical model, is applied to reveal the dominant factors on the heat transfer performance. Meanwhile, the effectiveness of the FHTP (*η*) is defined as the ratio between *Q_s_* and *Q_f_*. Thus, *Q_s_* and *η* can be expressed as the following:

(5) $$\begin{array}{ccc} Q_{s} & = & \frac{\alpha_{s}\alpha_{a}\left(T_{i}-T_{a}\right)\cdot n\left(D+d\right)}{\alpha_{s}+\alpha_{a}}\cdot l\\ & & +\;c\rho \nu \cdot \frac{{\alpha_{s}}^{2}\left(T_{i}-T_{in}\right)+\alpha_{s}\alpha_{a}\left(T_{a}-T_{in}\right)}{{\left(\alpha_{s}+\alpha_{a}\right)}^{2}}\cdot \left(1-e^{-\frac{\left(\alpha_{s}+\alpha_{a}\right)\cdot n\left(D+d\right)}{c\rho \nu }\cdot l}\right) \end{array}$$

(6) $$\eta =\frac{Q_{s}}{Q_{f}}\;\times 100\%$$

where *α_s_* and *α_a_* have been given in Equations (3) and (4), respectively. This model incorporates four categories of parameters: 1) the geometric dimensions of the FHTP (*n, D, d, l*), 2) the thermophysical properties of the TPU laminated or thermal insulation fabric (*R_pi_*, *R_po_*, *R_t_*), 3) the conditions of the water being pumped during cold or hot modes (*T_in_* and *v*), 4) the ambient air conditions (*T_a_*). *n*, *D*, and *d* determine the width of fluid channel. *Q_s_* is proportional to *n(D+d)* (Figure S18, Supporting Information). Here, based on the size of experimental sample, *n(D+d)* is kept as constant. Besides, a reasonable channel length (*l*) can be found under different flow rates, based on the heat transfer model (**Figure** **3**A). *Q_s_* is almost linearly dependent on the channel length within 2 m. When the flow rates are 0.8, 1.2, and 1.6 L min^−1^, the lines nearly overlap, which is higher than the line of 0.4 L min^−1^ (the inset in Figure 3A). This difference is mainly caused by the distinct contact pressure and area ratio under various flow rates, as shown in Figure 2F. With the increase of channel length, the slope of *Q_s_* becomes gentler. Thus, it is necessary to design the FHTP with appropriate channel length. Moreover, in practice, *T_a_* can be regarded as a constant, which is 22 °C in the model. Meanwhile, according to Equation (4), *R_po_* and *R_t_* influence *α_a_*. *R_t_* is the primary parameter; *R_po_* is regarded as a constant, as the thermal resistance of thermal insulation fabric is higher one order of magnitude than that of TPU laminated fabric (Table S5, Supporting Information). Therefore, four parameters, *R_pi_*, *R_t_*, *T_in_*, and *v*, are the main factors that influence the performance of the FHTP. Equations (5) and (6) can be further expressed by Equations S36–S39 (Supporting Information) (Note S6 and Table S6, Supporting Information).

Orthogonal design is a popular method for minimizing the number of experiments in the multi‐factor studies by using an orthogonal array.^[^ ^20^ ^]^ Range analysis helps determine the sensitivity ranking of factors to the evaluation indexes and identify the optimal factor level based on orthogonal results.^[^ ^21^ ^]^ In this study, orthogonal design and range analysis are employed to quantitatively reveal the sensitivity of the four factors (*R_pi_*, *R_t_*, *T_in_*, and *v*) on the heat transfer performance of the fluidic fabric. *Q_s_* and *η* is chosen as the evaluation index of heat transfer performance. The different levels of *R_pi_* and *R_t_* are chosen, based on the measurement of the commercial TPU laminated fabrics and thermal insulation fabrics (Table S5, Supporting Information). The factors and levels of the orthogonal design are shown in Table S7 (Supporting Information). The combination of different factors and levels is listed based on L_16_ (44) orthogonal table as well as the results of *Q_s_* and *η* as the evaluation index (Table S8, Supporting Information). *R_pi_* ranged from 0.002 to 0.006 m^2^ K W^−1^ affects slightly on the *Q_s_* and *η* value (Figure 3B). *Q_s_* keeps almost unchanged, when *R_t_* in the range of 0–0.07 m^2^ K W^−1^; whereas *η* rises gradually, when *R_t_* increases from 0 to 0.05 m^2^ K W^−1^ and keeps unchanged when *R_t_* further increase (Figure 3C). Thus, to select the appropriate materials for fabricating comfort and effective FHTP, *R_t_* should not exceed 0.05 m^2^ K W^−1^. Furthermore, *η* drops significantly when *T_in_* cools from 20 to 5 °C. The average *η* falls to ≈82% when *T_in_* is 5 °C (Figure 3D). The reason for the decrease of *η* is that the flowing water with lower temperature can absorb more heat from the surroundings in room temperature. Therefore, it is quite important to conduct thermal insulation treatment on the surface of the FHTP, especially under the large temperature difference between flowing water and the surroundings. Meanwhile, there is a negative linear dependence between *Q_s_* and *T_in_*. Figure 3E indicates that both *Q_s_* and *η* obtain the maximum when the flow rate is 0.8 L min^−1^. As we discussed in the former section, when flow rate increases from 0.8 to 1.6 L min^−1^, the gradual increase of contact pressure and the decrease of contact area ratio lead to the slight reduction of *Q_s_*. As shown in Figure 3F, the importance of these four variables is compared via calculating the difference between the maximum and minimum of the average *Q_s_* and *η* in Figure 3B–E. As a result, inlet water temperature and the flow rate are the primary and secondary factors on both *Q_s_* and *η*. The thermal resistance of insulation fabric is also a key parameter in obtaining a high *η*. However, the resistance of inner fabric of the FHTP in the range of 0.002–0.006 m^2^ K W^−1^ has a slight influence on both *Q_s_* and *η*.

### 2.4 Human Wearing Trial for Cold and Hot Therapy

**Figure 4:**
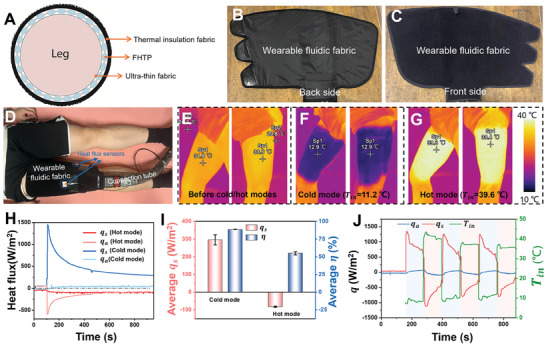
One wearable fluidic fabric for thigh and its heat transfer performance. A) The composite fabric structure of the wearable fluidic fabric from the cross‐sectional view: an outer thermal insulation fabric layer, a middle FHTP, and an ultra‐thin inner fabric in contact with the skin. This fluidic fabric is fabricated based on the study in the above section. B,C) Photographs of the back and front side of the wearable fluidic fabric, respectively. The Velcro fastener is applied to assist users to wear the FHTP. D) Photograph of the fluidic fabric worn on a participant's right thigh. The fluidic fabric is linked to the control machine via connection tube. E–G) IR images of the thigh before and after 15 min cold and hot therapy with an inlet water temperature of 11.2 and 39.6 °C, respectively. Sp1 indicates the surface temperature of skin. To minimize heat loss while capturing IR images after detaching the fluidic fabric, the wearer detaches the fabric themselves while the fluid is still circulating. Then, the wearer stands up and positions their inner and outer thigh sequentially in front of the IR camera, which is pre‐set and ready to capture images. The entire process, from detaching the fluidic fabric to capturing the IR images, takes ≈5–10 s. H) Heat flux during both cold and hot therapies. I) Heat flux between skin and fluid (*q_s_*) and effectiveness (*η*) of fluidic fabric during cold and hot therapies. J) *q_s_*, *q_a_*, and *T_in_* versus time when the fluidic fabric was worn on the thigh during rapid contrast therapy. The fluidic fabric was connected to our machine running a customized program of three cooling/heating cycles. Each cycle consists of 2 min in cold mode followed by 2 min in hot mode.

Based on the above analysis, a wearable fluidic fabric for the usage on thigh is fabricated. The composite fabric structure of the wearable fluidic fabric from the cross‐sectional view: an outer thermal insulation fabric layer, a middle FHTP, and an ultra‐thin inner fabric in contact with the skin. (**Figure** **4**A). Its channel length is much shorter than that of the previous FHTP used for the lower limb (Figure S3, Supporting Information). A layer of thermal insulation fabric was covered on the surface of the FHTP to reduce the heat transfer between fluid and the surroundings. Meanwhile, a Velcro fastener was adopted to assist the wearable fluidic fabric being worn. The inner and outer sides of the wearable fluidic fabric are exhibited in Figure 4B,C, respectively. Figure 4D shows the fluidic fabric is worn on a right thigh of a participant at the room temperature of ≈20.8 °C. The surface skin temperature of right thigh from both inner and outer sides were captured by IR camera before and after cold/hot modes (Figure 4E–G). The entire thigh can be uniformly cooled or warmed after cold or hot mode. The initial skin temperature of inner thigh is 31.8 °C and outer thigh is 33.6 °C (Figure 4E) After 15‐min cold therapy, the surface temperature of thigh is uniformly cooled down to 12.9 °C with *T_in_* of 11.2 °C (Figure 4F). After 15‐min hot therapy, the surface temperature of thigh is uniformly warm up to ≈39.3 °C with *T_in_* of 39.6 °C (Figure 4G). The small difference between skin temperature and inlet water temperature suggests a satisfactory skin cooling and heating performance of the wearable fluidic fabric.

The heat flux (*q*) between the skin and the wearable fluidic fabric, wearable fluidic fabric, and surroundings during cold and hot therapies were continuously recorded. (Figure 4H). The heat flow direction from the skin or surroundings to the wearable fluidic fabric is considered as positive. During the cold therapy, the cooling rate between skin and fluidic fabric, *q_s_*, instantly reaches its maximum of 1445 W m^−2^ at the beginning, which gradually decreases due to the decrease of the temperature difference between the skin and the flowing water. The *q_s_* is nearly stable at 321 W m^−2^ at the end of cold therapy. Similarly, in hot therapy, the absolute value of *q_s_* obtains the maximum of 586 W m^−2^ and gradually falls to 86 W m^−2^, which is smaller than that in cold therapy. However, the absolute value of heat flux (*q_a_*) in hot therapy is ≈78 W m^−2^, larger than that in cold therapy (≈39 W m^−2^). Figure 4I depicts the averaged heat flux and effectiveness in both cold and hot therapy. The near‐steady‐state cooling rate of this fluidic fabric in cold therapy with *T_in_* of ≈11 °C is 296 W m^−2^. Also, the effectiveness of *η* is up to 89% in cold therapy, indicating extremely high thermal utilization. However, the *η* in hot therapy is only 55%. The *η* depends on the ratio of the temperature difference between the surroundings and fluid, (*T_a_* – *T_f_*), to that between the skin and fluid, (*T_i_* – *T_f_*). A higher ratio results in lower effectiveness. Please see the detailed information in Note S7 (Supporting Information). Therefore, thermal insulation fabric with higher thermal resistance is required in hot therapy. Furthermore, the fluidic fabric was worn on the thigh and connected to our control machine to measure the heat transfer performance of our fluidic fabric during rapid contrast therapy (Figure 4J). This customized therapy consists of three cooling/heating cycles, each including 2 min in cold mode followed by 2 min in hot mode. The *q_s_* instantly exceeded 1000 W m^−2^ at the onset of each mode, demonstrating a rapid transition between cold and hot modes. Meanwhile, the *q_a_* showed minimal variation throughout the therapy, indicating a high heat transfer effectiveness during cold/hot modes.

## 3 Conclusion

In this work, a wearable fluidic fabric with a high heat transfer coefficient of up to 98.5 W m^−2^ K^−1^ has been developed. This fluidic fabric is suitable for rapid contrast therapy, as it achieves 92% efficiency of traditional cold‐water immersion. The core layer of wearable fluidic fabric, the FHTP was designed in a network pattern and fabricated with two pieces of TPU laminated fabrics by an easy and large scalable two‐step process. It can switch between cold and hot modes (5–40 °C) within 10 s over an effective area of 0.3 m^2^. A mathematical model of the FHTP considering the contact condition arising from the FHTP deformation was developed and verified with experiments. Based on the verified model, four key factors — the thermal resistance of inner fabric and thermal insulation fabric, as well as the temperature and flow rate of inlet water — of ten parameters were identified, which determined the performance of the FHTP in terms of heat transfer rate and effectiveness. Among them, the temperature and flow rate of inlet water are the two dominant factors. One wearable fluidic fabric optimized for thigh usage is fabricated, consisting of the FHTP as core layer, an outer thermal insulation fabric layer, and an inner ultra‐thin water‐proof fabric. This wearable fluidic fabric showed excellent heat transfer performance. During 15‐min cold therapy, the maximum heat flux between skin and fluidic fabric reached over 1445 W m^−2^. The near‐steady‐state heat flux and effectiveness of the fluidic fabric were as high as 296 W m^−2^ and 89%, respectively. This fluidic fabric has potential applications in diverse scenarios, such as personal thermal management systems, fire‐protection suits, external cryotherapy in medical settings, and providing tactile and temperature sensations in virtual reality.

## 4 Experimental Section

### Materials and Fabrication of Textile‐based Fluid Channel

The laminated fabrics were composed of two layers: TPU and nylon/polyester fabrics in plain weave (Figure S19, Supporting Information). The yarn counts, warp/weft yarn density, fabric weight, and thickness are listed in Table S9 (Supporting Information). Fluid channels were constructed by sticking two pieces of TPU laminated fabrics along designed lines and dots with high‐frequency dielectric welding technique. During the welding process of lower limb‐FHTP and the rectangle FHTP, the electrode temperature and welding pressure were set to be 130 °C and 0.2 Mpa, respectively. Figure S20 (Supporting Information) shows the surface and cross‐sectional images of the FHTP, indicating the formation of textile‐based fluidic channel.

### Thermal Analysis and Heat Transfer Model

The actual heat transfer pathway between a body, the FHTP, and the surroundings can be highly complex. To facilitate analysis and meet the requirements of practical engineering applications, it was essential to make certain assumptions regarding the heat transfer process.

The assumptions include:

1. The heat transfer was in a steady state.
2. Human body remained static, the convection type between the FHTP and surroundings was natural convection.
3. The heat transfer between the body and the surroundings was ignored.
4. The respiratory and evaporation heat transfer of body was ignored.
5. Conductive heat transfer between skin and the FHTP was only along the through‐plane direction.
6. Condensation heat transfer was ignored.

Based on the above assumptions, the heat from the body and the surroundings was entirely transferred to the flowing water in the FHTP during the cold mode. Conversely, during the hot mode, the heat from the flowing water in the FHTP was completely transferred to the body and surrounds. This heat transfer pathway during cold and hot modes is illustrated in Figure 2C. Consequently, the energy balance equation for the body‐FHTP‐surroundings system can be simplified as follows:

(7) $$Q_{f}=Q_{s}+Q_{a}$$

The mathematical heat transfer model was established based on this energy balance equation and the detailed deviation has been introduced in Notes S1 and S3 (Supporting Information).

### Experimental Setup and Testing Procedures for Experimental Validation

An experimental set‐up was constructed to validate the heat transfer model, which includes two single thermostatic water circulation systems, one can supply constant inlet water of 35 ± 0.2 °C pumping into the curved platform. The other can supply the constant cold water into the FHTP (Figure S9, Supporting Information). Thermostatic curved platform was designed to simulate the curvature of human thigh (Figure S10A, Supporting Information) and showed a uniform surface temperature when a constant water temperature of 35 °C was circulated through the internal channel of the platform (Figure S10B, Supporting Information). One FHTP for the experimental validation was rectangular (Figure S5B, Supporting Information), whose dimensions are listed in Table S10 (Supporting Information). The FHTP had three inlets and three outlets to obtain uniform flow within the channel (Figure S11A–C, Supporting Information). Thermal insulation foams with thermal conductivity of 0.014 W m^−1^ K^−1^ covered the ends of the double sides (beyond the network channel) of the FHTP to minimize heat transferring from the FHTP to the curved platform and the surroundings (Figure S11D,E, Supporting Information).

The silicone tube connecting the FHTP to the water tank was wrapped by thermal insulation foam with thickness of 5 mm. The inlet water at constant temperature was supplied by a JULABO immersion circulator (ED v.2, JULABO GmbH Germany). The test was conducted in a laboratory at constant temperature of 22 °C and humidity of 65%. The variation of ambient temperature was less than ± 0.5 °C.

The test procedure was listed as follows:

1. The hot water in temperature of 35 ± 0.2 °C was pumped into curved platform.
2. To test *Q_s_*, a heat flux sensor with size of 5 × 5 mm^2^ was fixed onto the surface of the curved platform using double‐side thermally conductive tape.
3. The FHTP was wrapped around the surface of the curved platform via Velcro fasteners (Figure S11F, Supporting Information).
4. To test *Q_a_*, a heat flux sensor (1 × 1 mm^2^) was fixed on the outer surface of the FHTP.
5. After both the heat flux and temperature data became stable, water was pumped into the FHTP.
6. The duration of each measurement was over 15 min to ensure the accuracy of the experimental data.

### Characterization

The optical images were captured by the optical microscope (LEICA M165C, Danaher Corporation). The thickness of TPU laminated fabric was measured by a thickness gauge (BG1110‐1‐04). The temperature was measured using the K‐type thermocouples and recorded by Anbat AT4516 (Applent Instruments Ltd.). The heat flux was recorded by heat flux sensors (FHF 05 series, Hukseflux company) and collected by a read‐out datalogger (LI‐19C). Thermal images and videos were captured by the thermal imaging camera (Fluke Ti400U Thermal Imager, precision: ± 2 °C or 2%, temperature resolution: 0.1 °C). The pressure of the FHTP on the surface of the curved platform under different water flow rates was measured using a thin‐film FlexiForce sensor (Tekscan, Inc.). The contact area ratio under different water flow rates was obtained via painting dyed sodium alginate solution on the inner fabric of the FHTP and calculated the area of solution absorbed by A4 paper (Figure S14, Supporting Information).

### Fabrication of Wearable Fluidic Fabric

The wearable fluidic fabric was designed for thigh's usage and constructed using a composite fabric structure comprising three distinct layers. FHTP was the core layer. A layer of thermal insulation fabric and an ultra‐thin fabric, both of identical dimensions to the FHTP, were positioned on either side of the FHTP. The thigh‐FHTP was fabricated via fabric cutting and on‐step welding. These three layers were then sewn together along the edges to ensure structural integrity. To facilitate ease of wear, a Velcro fastener was incorporated into the design.

### Human Trial Test Procedures

To evaluate the heat transfer coefficient (*h*) of the wearable fluidic fabric in comparison to cold water immersion, two heat flux sensors in size of 10  ×  10 mm^2^, were affixed to the outer surface of the fluidic fabric at the inlet and outlet positions using thermally conductive tape. The heat flux (*q_a_*) was determined by averaging the data collected from these two sensors. The heat transfer rate (*Q_f_*) was calculated based on the water flow rate and the temperature difference between the inlet and outlet water. The thigh skin area covered by the wearable fluidic fabric (*S*) was ≈0.14 m^2^. The fluid temperature within the FHTP was calculated as the average of the inlet (*T_in_*) and outlet (*T_out_*) water temperatures, while the average skin temperature (*T_s_*) was obtained through IR images. Consequently, the heat transfer coefficient was calculated using the formula:

(8) $$h=\left(Q_{f}/S-q_{a}\right)\;/\left(\left(T_{in}+T_{out}\right)/2-T_{s}\right)$$

To continuously monitor heat transfer rate between skin and fluidic fabric (*q_s_*), a larger heat flux sensor measuring 50 × 50 mm^2^ was attached to the skin surface with thermally conductive tape. Simultaneously, a 10 × 10 mm^2^ heat flux sensor was positioned on the outer surface of the fluidic fabric directly above the larger sensor. The effectiveness (*η*) of the heat transfer was calculated using the following equation:

(9) $$\eta =q_{s}\;/\left(q_{s}+q_{a}\right)\times 100\%$$

For both tests, the inlet water temperature was maintained at ≈10 °C, and the flow rate was kept constant at 4 L min^−1^. Each test was conducted for a duration of 15 min and repeated three times.

## Conflict of Interest

The authors declare no conflict of interest.

## Author Contributions

X.T. and J.Y. conceived the concept of the FHTP. X.T. obtained funding and supervised the project. J.Y. contributed to the establishment and experimental validation of the heat transfer model, conducted human trial tests, and drafted the manuscript. Y.X. was responsible for the fabrication and production of the machine and connector tube. J.P. assisted in the establishment of the heat transfer model. J.Y. fabricated the FHTPs in serpentine and network channel patterns and, along with Y.X., conducted the evaluation tests. G.L. fabricated the lower limb FHTP and assisted with the FTIR test. M.L. confirmed the outline of the thigh‐FHTP, designed its wrap, and drew an illustration of the wearable system. J.Y., Y.X., G.L., S.T., and M.Z. equally contributed to the pattern design and optimization of the thigh‐FHTP. J.Y. was responsible for the large‐scale production of wearable fluidic fabric. L.Z. and X.T. reviewed and edited the manuscript.

## Supporting information



Supporting Information

Supplemental Movie 1

## Data Availability

The data that support the findings of this study are available from the corresponding author upon reasonable request.
