# Supplementary material for: Wearable Fluidic Fabric with Excellent Heat Transfer Performance for Sports Recovery
Source: Adv Sci (Weinh). 2025 Jan 7;12(8):2411691. doi: 10.1002/advs.202411691 (PMC11848550; doi:10.1002/advs.202411691)
Supplement: Supplementary file 1 — Supporting Information [file ADVS-12-2411691-s002.docx]

Supporting Information

Wearable Fluidic Fabric with Excellent Heat Transfer Performance for Sports Recovery

*Jing Yang^a,b^, Ying Xiong^a,b^, Jinli Piao^a,b^, Manyui Leung^a,b^, Guosai Liu^a,b^, Mingyue Zhu^a,b^, Shengyang Tang^a,b^, Lisha Zhang^a,b,^*, Xiaoming Tao^a,b,^**

^a^ Research Institute for Intelligent Wearable Systems, The Hong Kong Polytechnic University, Kowloon, Hong Kong, 999077, China

^b^ School of Fashion and Textiles, The Hong Kong Polytechnic University, Kowloon, Hong Kong, 999077, China

Corresponding author(s):

Email: xiao-ming.tao@polyu.edu.hk; lisa-lisa.zhang@polyu.edu.hk

This supplementary information contains

Supplementary Figures 1-20

Supplementary Tables 1-11

Supplementary Note 1: Deviation of heat transfer model

Supplementary Note 2: Justification for neglecting heat transfer between dots and skin

Supplementary Note 3: Calculation on the thermal resistance

Supplementary Note 4: The value of correction factor ($f_{int}$)

Supplementary Note 5: Experimental validation under various inlet water temperature ($T_{in}$)

Supplementary Note 6: Deviation of $Q_{s}$ and $\eta$ with only four parameters ($R_{pi}$, $R_{t}$, $T_{in}$ and $\nu$)

Supplementary Note 7: The correlation of the effectiveness ($\eta$) related to the temperature difference

Supplementary Movie:

File Name: Supplementary Movie 1

Description: The IR video about the surface temperature change of the FHTP in transition between cold mode and hot modes. Cold mode: ~5 ℃ for 1 min, and hot mode: ~40 ℃ for 2 min.

-

**Supplementary Figures**


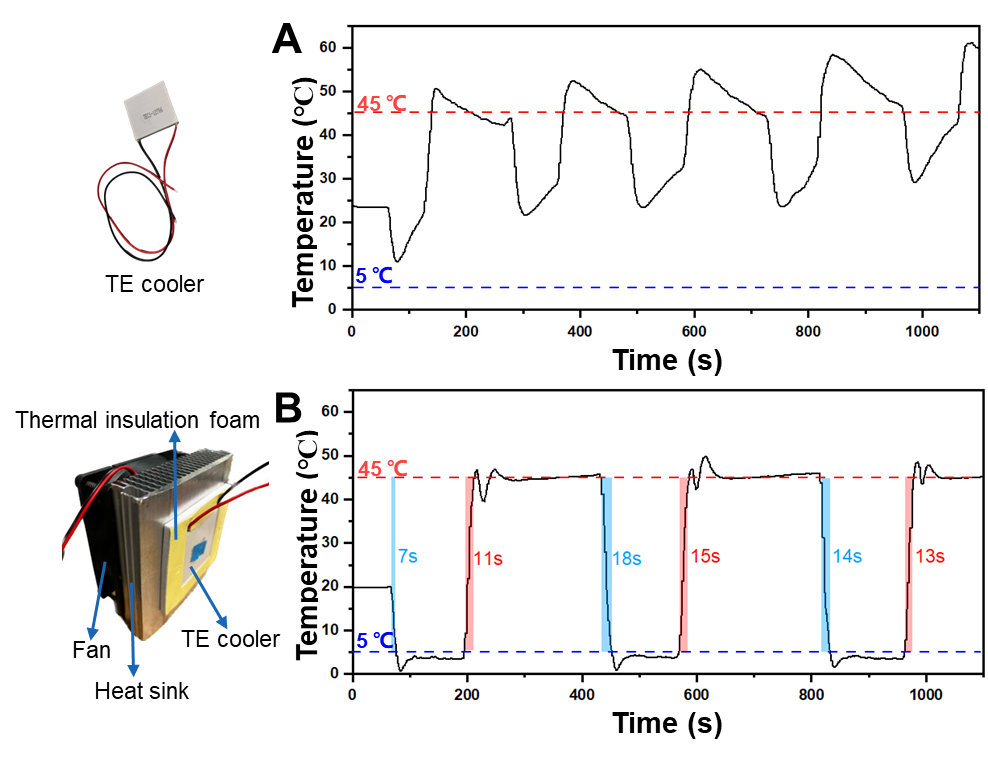


1. The cooling and heating performance of commercial thermoelectric (TE) cooler equipped with/without heat dissipation unit. A) Temperature on the surface of the TE cooler without fans nor heat sinks over time. The dimensions and weight of the cooler is 40$\times$40 $\times$4 mm^3^ and 23.5 g, respectively. TE cooler cannot even reach to 5 ℃ during cooling/heating cycles. B) Temperature on the surface of the TE cooler equipped with heat dissipation unit over time. The heat dissipation units included a fan and a heat sink. The air flow rate of the fan was 202 CFM, and the dimensions of the aluminum heat sink was 90$\times$90$\times$25 mm^3^. The input power of fan is ~16 W, and the total weight of TE cooler and heat dissipation unit is 450 g. The duration switched between 5 ℃ and 45 ℃ is slighter than 10 s, close to the requirements on the rapid transition between cold and hot modes for rapid contrast therapy. The input power of TE coolers in both A) and B) was consistent. Thus, although the TE cooler can switch between cooling and heating modes rapidly, the extra heat dissipation unit greatly increases the total weight and power consumption.


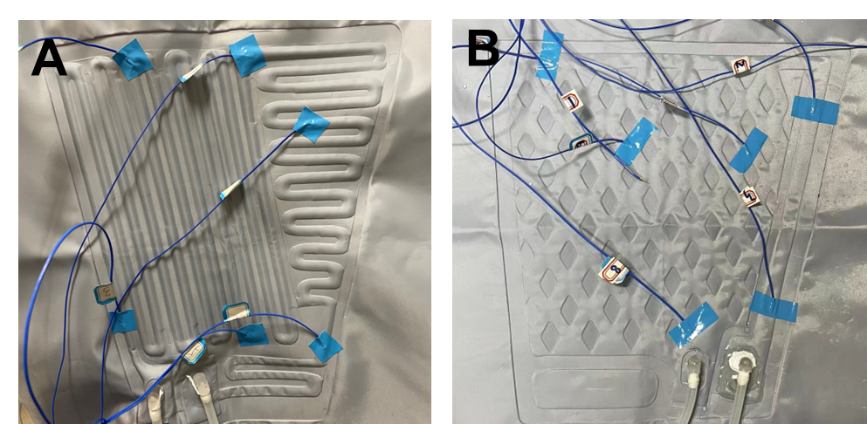


1. The FHTPs are fabricated in two fluid channel patterns. A) Serpentine channel pattern. B) Network channel pattern. To evaluate temperature uniformity, six k-type thermocouples were randomly placed on the surface of the FHTPs.


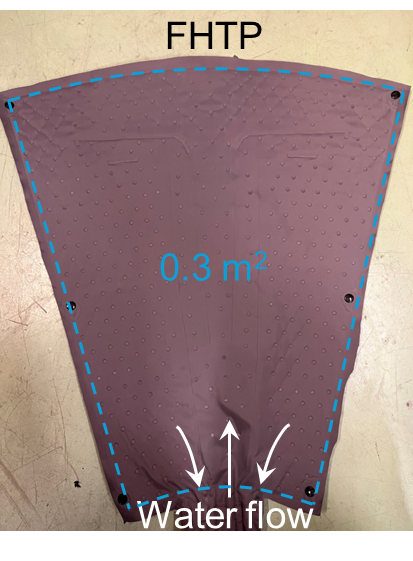


1. Photo of the FHTP for lower limb. The area enclosed by blue dotted line was the effective area for skin cooling/heating.


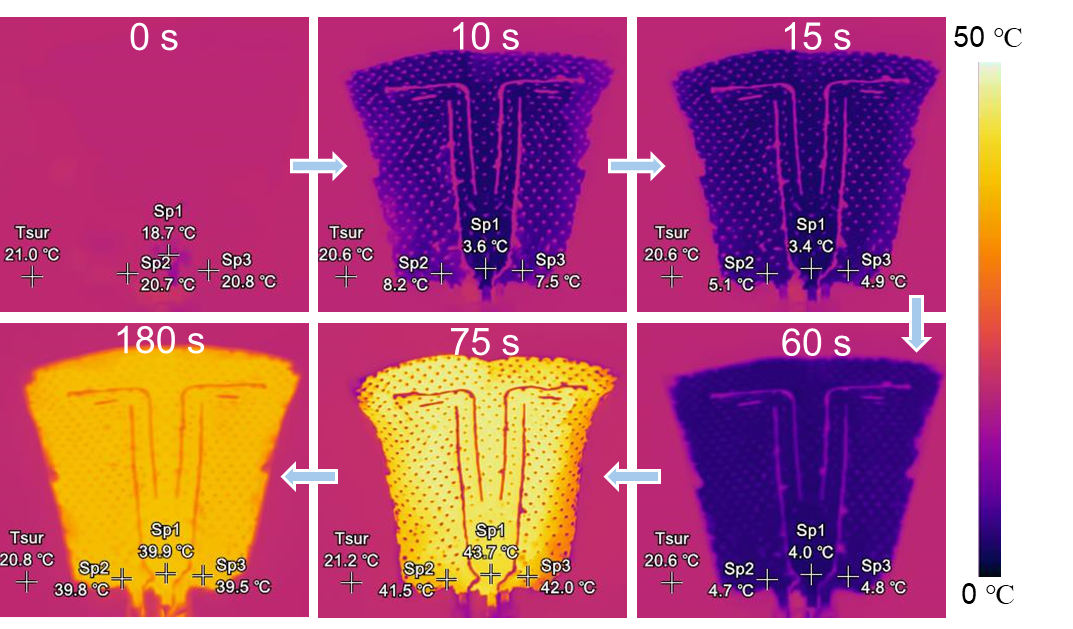


1. IR images at different moments during a cooling/heating cycle of the FTHP for lower limb.


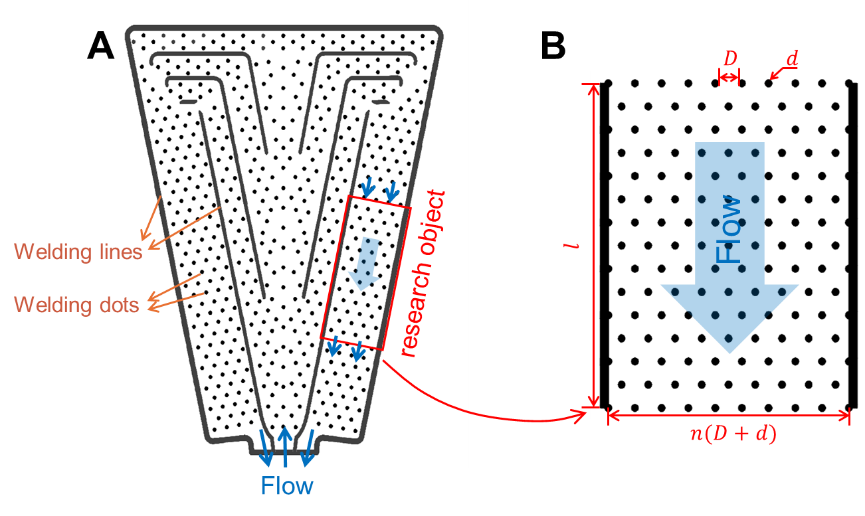


1. A) Network channel pattern of the FHTP for lower limb. B) Rectangle fluid channel as the research object in the heat transfer model. Dark solid dots and lines represent the welding zone. $l$ is the channel length. $d$ is the diameter of welding dot. $D$ is the distance between two adjacent dots. $n(D+d)$ is the channel width.


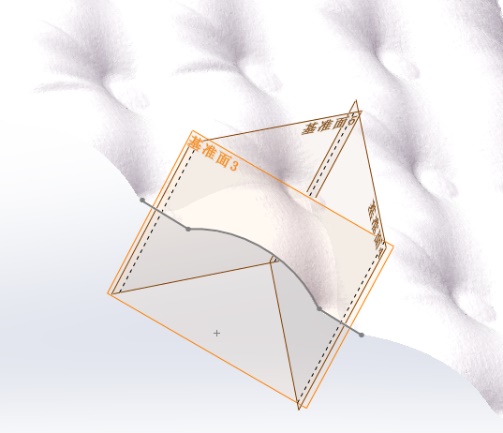


1. The curve between two adjacent welding dots of the inflated FHTP under air pressure of 8 KPa.


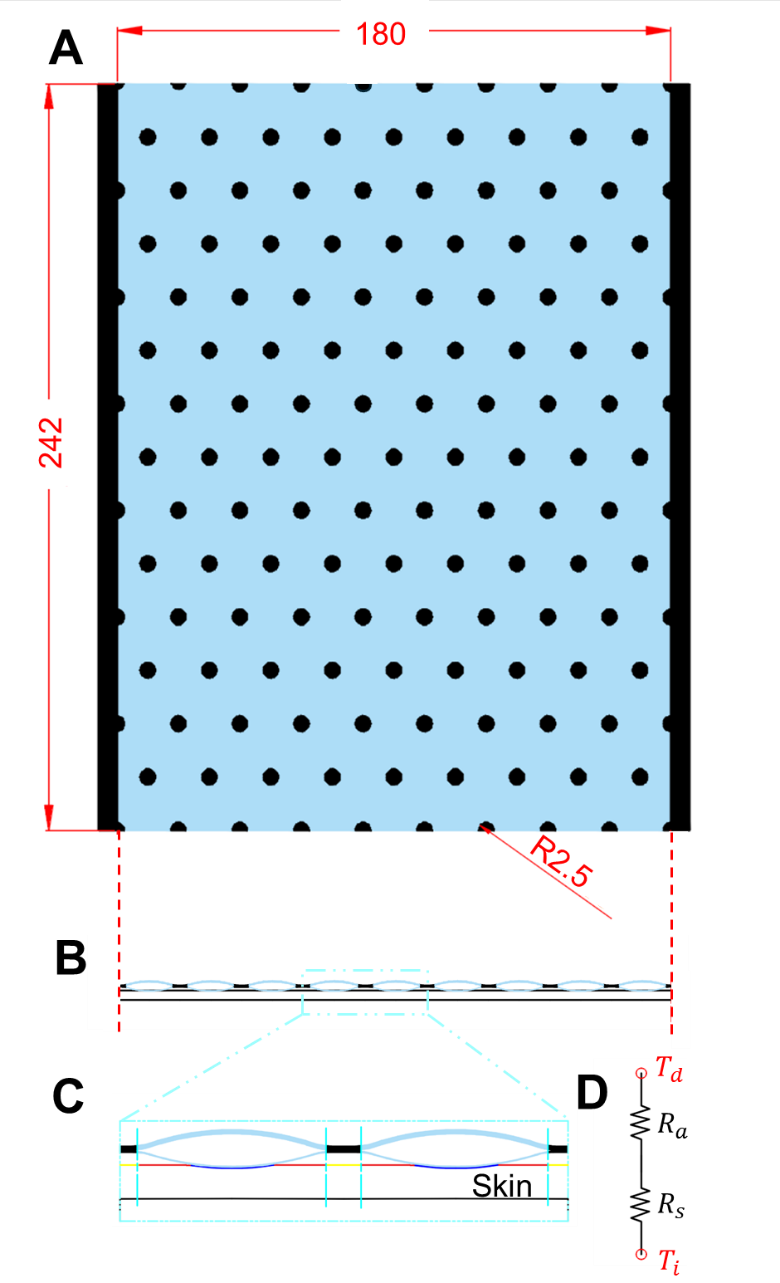


1. A) Top view of the two-dimensional illustration of the rectangular FHTP. The dark areas indicate the welding zones, including lines and dots, while the grey-blue areas represent the fluidic channels. B) Cross-sectional view of the FHTP. C) Enlarged detail of B), showing the yellow lines as the non-contact areas between welding dots and skin, red lines as the non-contact areas between fluidic channels and skin, and blue lines as the contact areas between fluidic channels and skin. D) The equivalent thermal resistance network between the dots and skin.


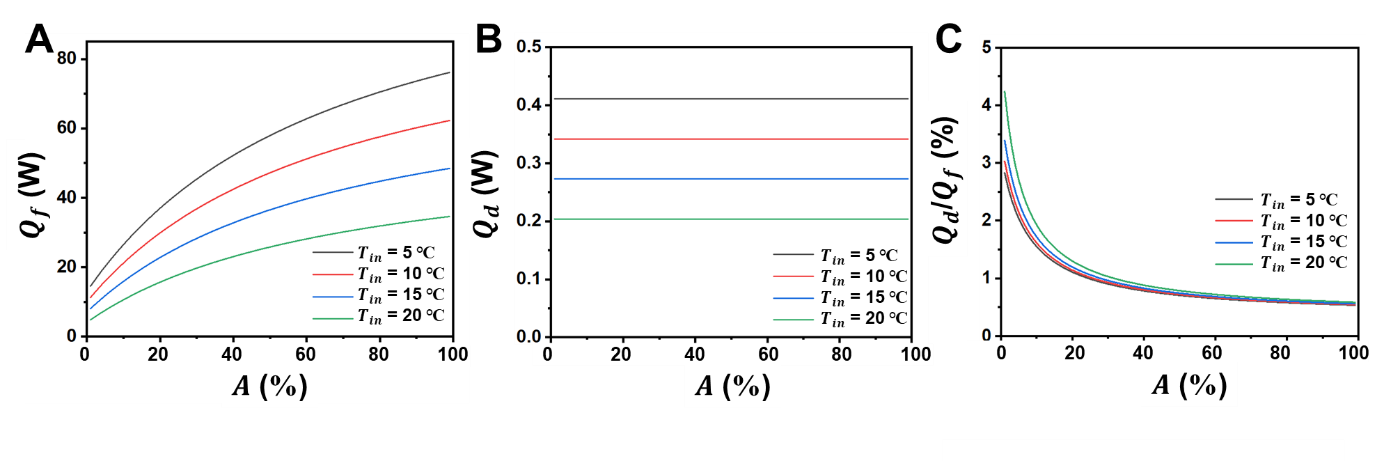


1. The analytical results of the heat transfer rate of fluid ($Q_{f}$) and heat transfer rate between dots and skin ($Q_{d}$). Effect of contact area ratio on the $Q_{f}$ A), $Q_{d}$ B) and $Q_{d}$/$Q_{f}\times$ 100% C) under four different inlet water temperature.


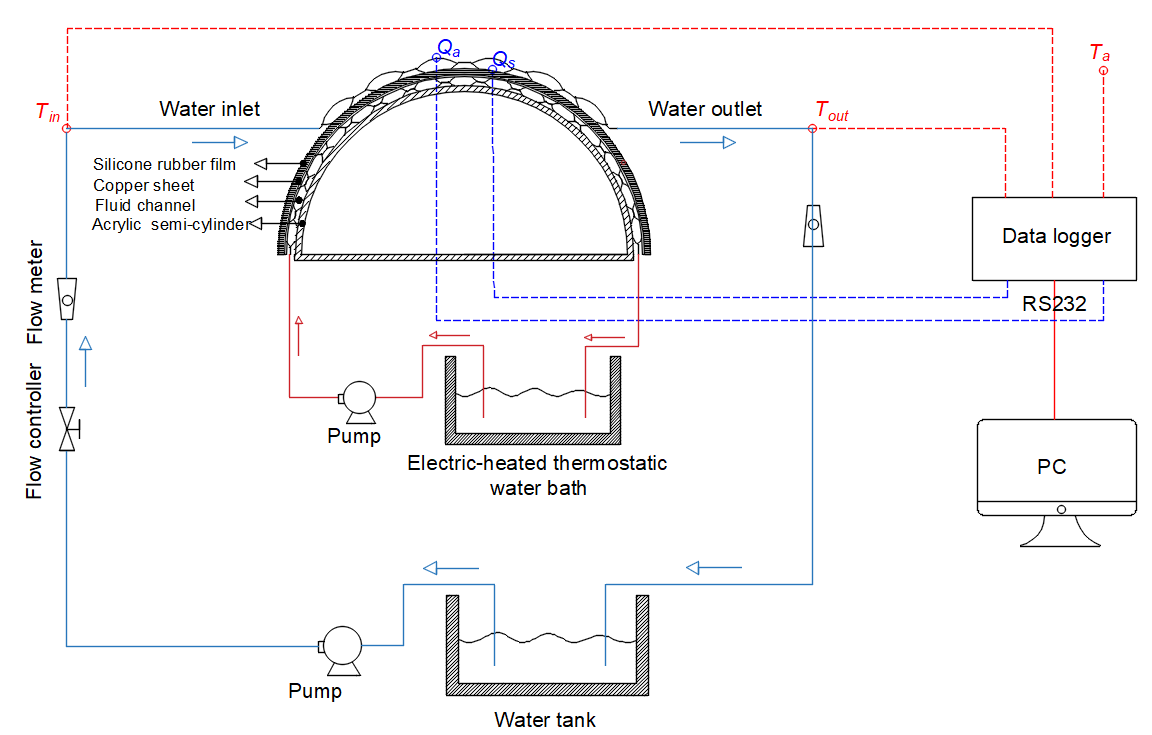


1. Schematic of the experimental setup for validating the established heat transfer model. The setup includes thermostatically controlled water circulation systems for curved platform (red solid lines) and the FHTP (blue solid lines). The curved platform is composed of silicone, copper sheet, fluid channel and acrylic, which are stacked together via double-side thermal conductive tape. Red and blue dotted lines represent data collection paths of temperature and heat flux, respectively.


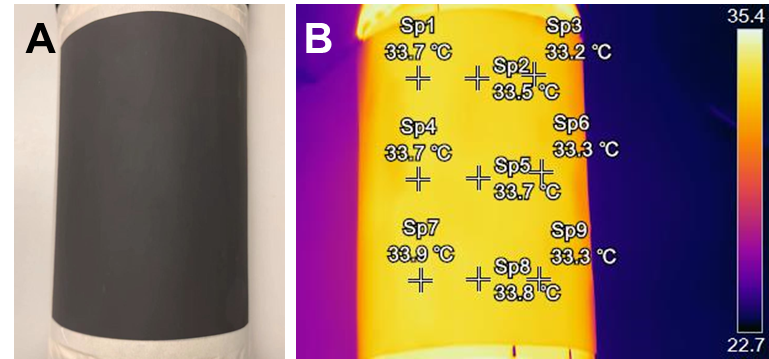


1. A) Photo of the curved platform mimicking a human leg. B) IR image of the surface temperature of the curved platform.


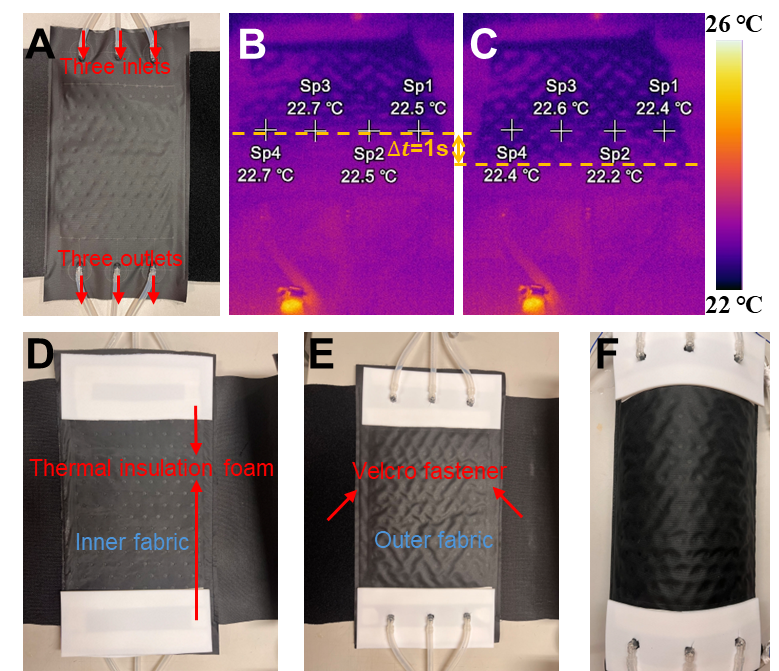


1. The FHTP for experimental validation. A) The FHTP is equipped with three inlets and three outlets. B) and C) IR images of water flowing in the FHTP. The interval ($\Delta t$) between the images being captured was 1s. Temperature along the orange dashed lines indicates water uniformly flowing in the FHTP. D) and E) The inner and outer fabrics of the FHTP with thermal insulation foam stuck near the inlets and outlets. F) The FHTP was wrapped on the surface of the curved platform via Velcro fastener.


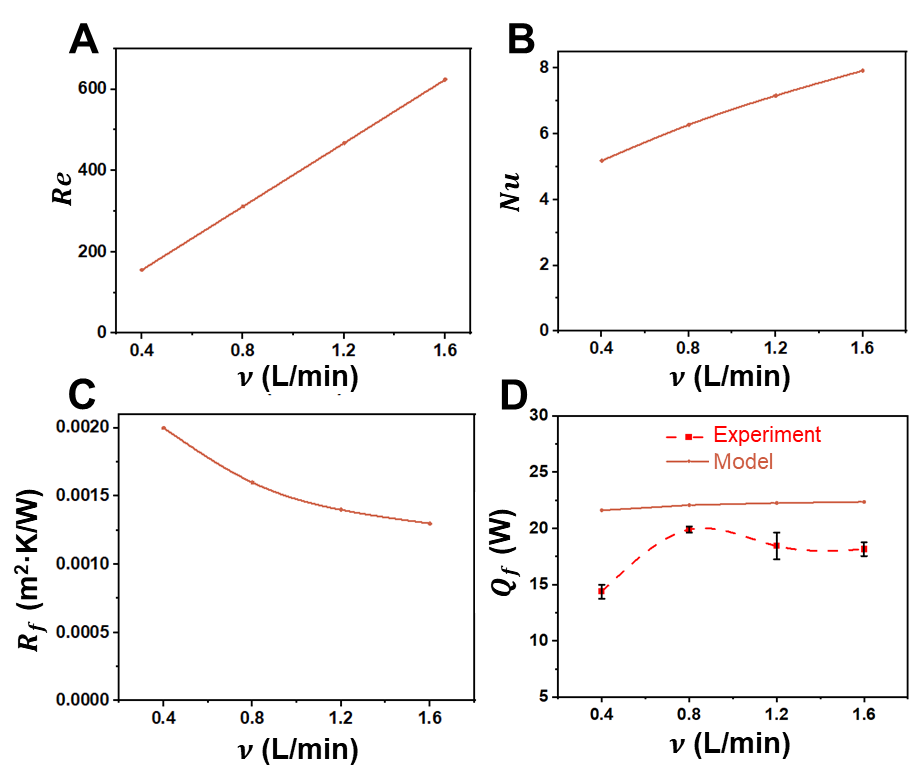


1. A-C)$Re$, $Nu$ and $R_{f}$ obtained by modelling under various flow rates (*v*). D) The experimental and analytical results of $Q_{f}$ under different *v.*


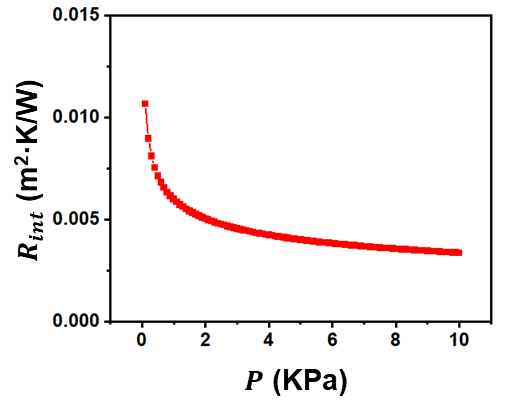


1. The relationship between thermal contact resistance ($R_{int}$) and contact pressure ($P$).


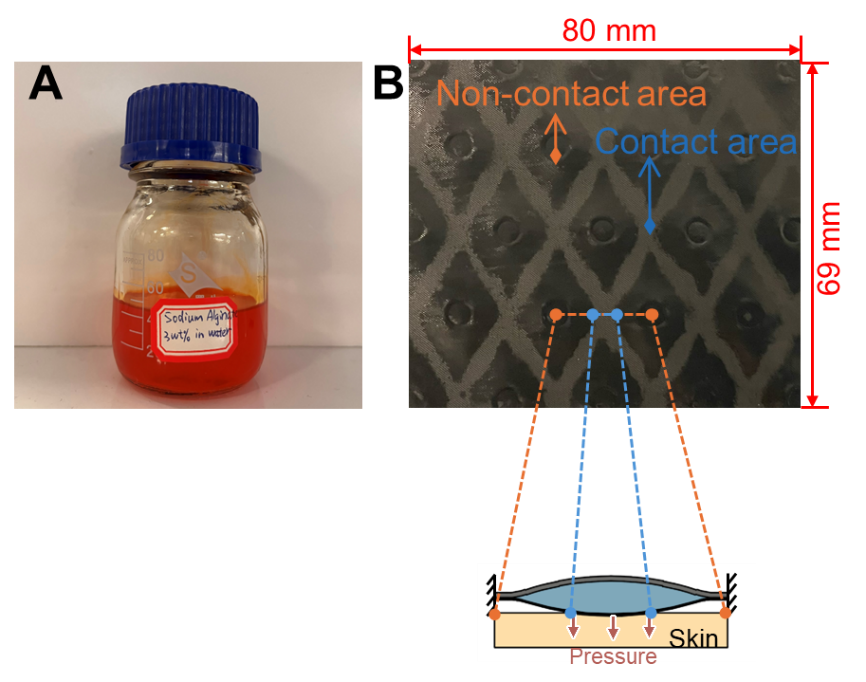


1. Measurement of the contact area ratio. A) Photo of a bottle of 3 wt% sodium alginate solution. B) Photo of surface of the FHTP with sodium alginate solution and the correlation of the contact area between the experimental photo from the bottom view and the illustration from the cross-sectional view. Inner surface of the FHTP was painted evenly with the solution. An A4 paper was placed on the curved platform. Then, the FHTP was wrapped on the curved platform by Velcro fastener. The painted surface faced the A4 paper. Next, water went through the FHTP with flow rate of 0.8 L/min for 2 min. In this photo, the light grey network represents the contact area, for some solution on the surface of the FHTP has been transferred to the paper. Meanwhile, the dark grey diamonds are the non-contact area.


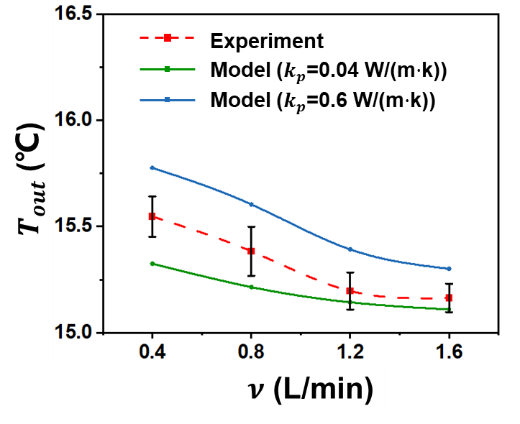


1. The experimental and analytical results of $T_{out}$ under different flow rates. Two model results of $T_{out}$ are derived when the thermal conductivity of the FHTP, assumed to be a homogeneous material, is set to 0.04 W/(m$\cdot$K) and 0.6 W/(m$\cdot$K), corresponding to the conductivities of inner fabric and flowing water, respectively.


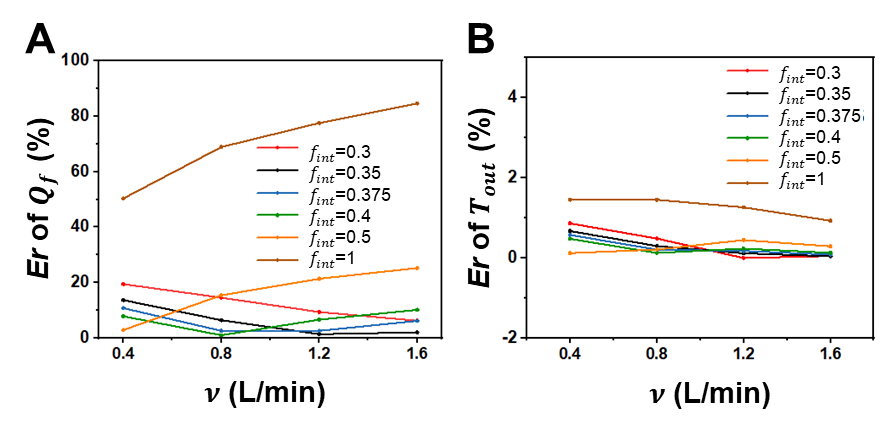


1. The $Er$ between analytical and experimental results on A) $Q_{f}$ and B) $T_{out}$ when correction factor $f_{int}$ is different.


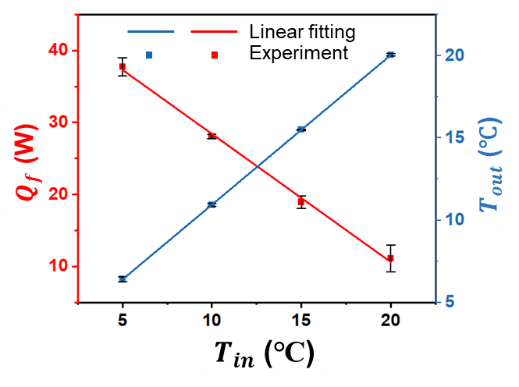


1. The linear fitting of experimental $Q_{f}$ and $T_{out}$ under various $T_{in}$. $Q_{f}=46.20-1.78{\cdot T}_{in}$ with $R^{2}=0.9967$. $T_{out}=1.85+0.91 {\cdot T}_{in}$ with $R^{2}=0.9999$.


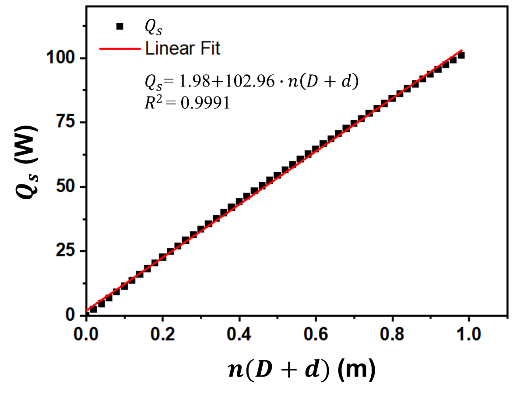


1. $Q_{s}$ as a function of $n(D+d)$ derived from the heat transfer model. $Q_{s}=1.98+102.96 \cdot n\left( D+d \right)$ with $R^{2}=0.9991$.


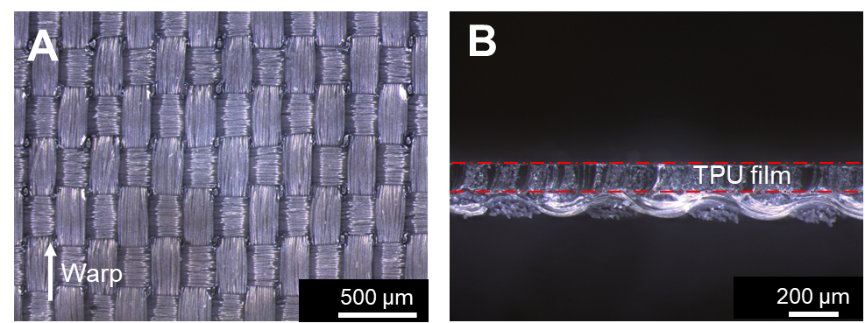


1. Optical images of TPU laminated fabric: A) surface; B) cross-section.


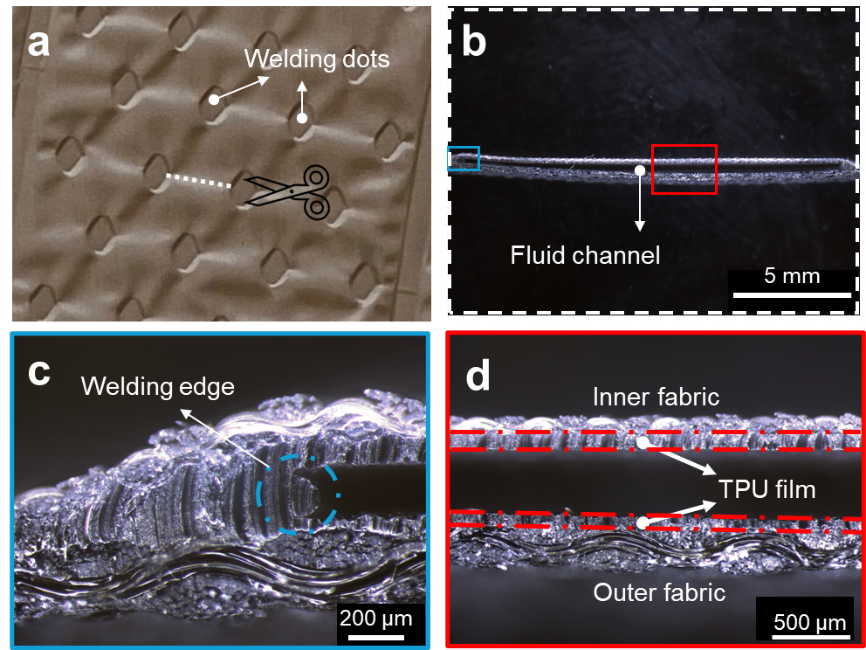


1. Surface and cross-sectional images of the FHTP. A) Photo of the FHTP with rhombus welding dots. B) Cross-sectional images of the fluid channel with an approximate width of 15 mm. C) Magnified image near the welding dot. D) Magnified image of the fluid channel.

**Supplementary Tables**

| Note: The data shown in blue is estimated from figures in the references. The data in red is calculated based on the data provided in the references. A ‘- ‘indicates available data from the references. $T_{is}$ represents the initial skin temperature before garment starts to work, and $T_{c}$ represents the cooling temperature of the garments, indicating the temperature difference of the skin before and after the garments is activated. | Thigh-worn fluidic fabric | | | | | Cooling vest | Spacesuit | Spacesuit | Cooling jacket | Cooling vest | Cooling vest | Commercial cooling vest | **Garments** | | 1. Comparison of the heat transfer performance among liquid cooling garments. |
| --- | --- | --- | --- | --- | --- | --- | --- | --- | --- | --- | --- | --- | --- | --- | --- |
|  | Ice water | | | | | Cold water | - | Microencapsulated PCM+water | TEC+water | Ice pack+water | TEC+water | Ice water | **Coolant** | |  |
|  | TPU laminated fabric with welding pattern | | | | | Tubes | Tubes | 46 pipes | Pipeline | Tubings | tube-based piping pattern | tube-based piping pattern | **Fluidic channel** | |  |
|  | Thigh | | | | | Torso | Torso, arms, legs | Upper arms, trunk, thighs | Torso | Torso | Torso | Torso | **Covered body parts** | |  |
|  | Human trial | | | | | Human trial | Human trial | Thermal manikin | Human trial | Simplified thermal manikin | Human trial | Thermal manikin | **Test subjects** | |  |
|  | Sports recovery | | | | | Hot environments | Space | Space | Thermal stress | Hot environments | Hot environments | Hot environments | **Potential application scenario** | |  |
|  |  |  | 0.3 | 0.42 | 0.42 | - | 0.7 | ~0.6 | - | 0.544 | 0.9 | - | L/min | $\boldsymbol{\nu}$ |  |
|  |  |  | 46.6 | 40.5 | 41.7 | 134-140 | 185 | 371 | 340.4 | - | 160.4 | - | W | $\boldsymbol{Q}_{\boldsymbol{f}}$ |  |
|  |  |  | - | - | - | 107 | - | - | - | - | - | ~70 | W | $\boldsymbol{Q}_{\boldsymbol{s}}$ |  |
|  | Thigh-worn fluidic fabric | | | | | Cooling vest | Spacesuit | Spacesuit | Cooling jacket | Cooling vest | Cooling vest | Commercial cooling vest | **Garments** | | **Continued** |
|  |  |  | 0.14 | 0.14 | 0.14 | 0.611 | 1.9 | ~ 1 | ~0.5 | 0.562 | 0.521 | ~0.5 | m^2^ | $\boldsymbol{S}$ |  |
|  |  |  | 333.1 | 289.2 | 297.6 | - | - | - | - | 243.2 | - | - | W/m^2^ | $\boldsymbol{q}_{\boldsymbol{f}}$ |  |
|  |  |  | 298.0 | 247.8 | 258.8 | - | - | - | - | 88.5 | - | - | W/m^2^ | $\boldsymbol{q}_{\boldsymbol{s}}$ |  |
|  |  |  | 35.0 | 41.3 | 38.8 | - | - | - | - | - | - | - | W/m^2^ | $\boldsymbol{q}_{\boldsymbol{a}}$ |  |
|  |  |  | 14.1 | 13.4 | 14.2 | 33 | 27.56 | 34 | ~33.9 | ~35 | - | 35 | ℃ | $\boldsymbol{T}_{\boldsymbol{s}}$ |  |
|  |  |  | 10.9 | 11.3 | 11.1 | 15 | 21 | 14.7 | 15.7 | ~22.5 | - | - | ℃ | $\boldsymbol{T}_{\boldsymbol{f}}$ |  |
|  |  |  | 20.4 | 21.4 | 20.8 | 30 | - | 18 | 30 | 45 | 39.2 | 35 | ℃ | $\boldsymbol{T}_{\boldsymbol{a}}$ |  |
|  | $\sigma$ | Average | 30.1 | 34.6 | 32.9 | 33 | - | 37 | ~34.3 | ~42.5 | - | 35 | ℃ | $\boldsymbol{T}_{\boldsymbol{is}}$ |  |
|  | 2.6 | 18.6 | 16 | 21.2 | 18.7 | 0 | - | 3 | 0.4 | 7.5 | - | 0 | ℃ | $\boldsymbol{T}_{\boldsymbol{c}}$ |  |
|  | 16.2 | 98.5 | 94.1 | 116.4 | 84.8 | 12.7 | 14.8 | 19.2 | 37.4 | 19.5 | - | - | W/(m^2^·K) | $\boldsymbol{h}$ |  |
|  | Our work | | | | | 26 | 25 | 24 | 23 | 22 | 21 | 20 |  | **Refs** |  |

1. Evaluation of the FHTPs in serpentine and network patterns during the cold mode

| **Evaluation** | | | **Channel patterns** | |
| --- | --- | --- | --- | --- |
|  |  |  | Serpentine | Network |
| **Temperature uniformity** | $T_{H}$*** | ℃ | 5.9 | 4.6 |
|  | $T_{L}$*** | ℃ | 4.0 | 2.9 |
|  | $T_{A}$*** | ℃ | 4.9 | 3.5 |
|  | $\sigma$* |  | 0.7 | 0.6 |
| **Water flow** | Water flow rate | L/min | 0.4 | 0.9 |
|  | Duration** | s | 7.0 | 4.0 |
|  | Volume | mL | ~80.0 | ~120.0 |

Notes: *$T_{H}$, $T_{L}$, $T_{A}$ and $\sigma$ represent the highest and lowest temperature detected by the six thermocouples, the average temperature, and the standard deviation, respectively. **Duration means the interval between water flowing in and out of the FHTP.

1. Experimental results of $\boldsymbol{Q}_{\boldsymbol{f}}$, $\boldsymbol{Q}_{\boldsymbol{s}}$and $\boldsymbol{Q}_{\boldsymbol{a}}$

| $\boldsymbol{\nu}$ | $\boldsymbol{Q}_{\boldsymbol{f}}$ | $\boldsymbol{\sigma}$ | $\boldsymbol{Q}_{\boldsymbol{s}}$ | $\boldsymbol{\sigma}$ | $\boldsymbol{Q}_{\boldsymbol{a}}$ | $\boldsymbol{\sigma}$ | ***Er*** |
| --- | --- | --- | --- | --- | --- | --- | --- |
| **L/min** | **W** |  | **W** |  | **W** |  | **%** |
| 0.40 | 14.37 | 0.62 | 13.34 | 2.71 | 2.68 | 6.82 | 10 |
| 0.80 | 19.89 | 0.27 | 19.76 | 0.58 | 2.46 | 11.16 | 10 |
| 1.20 | 18.44 | 1.18 | 16.88 | 0.68 | 2.46 | 9.21 | 5 |
| 1.60 | 18.15 | 0.61 | 17.23 | 0.71 | 2.41 | 9.57 | 8 |

Notes: $Er=\frac{\left| Q_{s}+Q_{a}-Q_{f} \right|}{\left( Q_{a}+Q_{s} \right)}\times100\%$. $\sigma$ represents the standard deviation.

1. Contact pressure and area ratio under various flow rates.

| $\boldsymbol{\nu}$ | **Contact pressure (KPa)** | | **Contact area ratio (%)** | |
| --- | --- | --- | --- | --- |
| **L/min** | **Average** | $\boldsymbol{\sigma}$ | **Average** | $\boldsymbol{\sigma}$ |
| 0.40 | 0.24 | 0.10 | 24.43 | 2.71 |
| 0.80 | 0.63 | 0.14 | 42.42 | 2.46 |
| 1.20 | 1.95 | 0.33 | 29.27 | 3.61 |
| 1.60 | 2.81 | 0.04 | 27.21 | 1.44 |

Notes: $\sigma$ represents the standard deviation.

1. The thickness and thermophysical parameters of the TPU laminated fabrics and the thermal insulation fabrics

| **No.** | **Thickness** | **Thermal Conductivity** | **Thermal Resistance** |
| --- | --- | --- | --- |
|  | **mm** | **W/(m·K)** | **m^2^·K/W** |
| **TPU laminated fabric** | | | |
| 1 | 0.12 | 0.039 | 0.003 |
| 2 | 0.09 | 0.042 | 0.002 |
| 3 | 0.14 | 0.046 | 0.003 |
| 4 | 0.16 | 0.050 | 0.003 |
| 5 | 0.16 | 0.056 | 0.003 |
| 6 | 0.19 | 0.058 | 0.003 |
| 7 | 0.19 | 0.056 | 0.003 |
| 8 | 0.17 | 0.052 | 0.003 |
| 9 | 0.16 | 0.057 | 0.003 |
| 10 | 0.45 | 0.072 | 0.006 |
| 11 | 0.50 | 0.092 | 0.005 |
| 12 | 0.18 | 0.027 | 0.007 |
| 13 | 0.08 | 0.003 | 0.025 |
| 14 | 0.18 | 0.078 | 0.002 |
| 15 | 0.20 | 0.079 | 0.003 |
| **Thermal insulation fabric** | | | |
| 1 | 2.16 | 0.038 | 0.060 |
| 2 | 1.61 | 0.052 | 0.030 |
| 3 | 2.20 | 0.031 | 0.070 |
| 4 | 2.71 | 0.041 | 0.070 |
| 5 | 2.80 | 0.052 | 0.050 |

1. Value of parameters as the constants in the model

|  | ***Symbol*** | ***Unit*** | ***Value*** |
| --- | --- | --- | --- |
| $R_{s}$ | $\delta_{s}$ | mm | 3.00 |
|  | $\lambda_{s}$ | W/(m·K) | 0.37 |
| $R_{a}$ | $\delta_{a}$ | mm | 4.00 |
|  | $\lambda_{a}$ | W/(m·K) | 0.023 |
| $R_{po}$ | $\delta_{po}$ | mm | 0.45 |
|  | $\lambda_{po}$ | W/(m·K) | 0.07 |
| $R_{int}$ | $\varepsilon_{1}$ | $\mu$m | 20.0 |
|  | $\varepsilon_{2}$ | $\mu$m | 6.38 |
|  | $\kappa_{1}$ | W/(m·K) | 0.37 |
|  | $\kappa_{2}$ | W/(m·K) | 0.60 |
|  | $G$ | KPa | 0.40 |
|  | $f_{int}$ |  | 0.375 |
| $R_{f}$ | $\rho$ | kg/m^3^ | 999 |
|  | $\mu$ | Pa·s | 1.14×10^-3^ |
|  | $c$ | J/(kg·K) | 4200 |
|  | $\lambda_{f}$ | W/(m·K) | 0.59 |
|  | $S_{ch}$ | mm^2^ | 30.00 |
|  | $p$ | mm | 33.00 |
| $R_{conv}$ | $h_{conv}$ | W/(m^2^·K) | 4.00 |
|  | $f_{cl}$ |  | 1.01 |
| $R_{r}$ | $h_{r}$ | W/(m^2^·K) | 4.70 |
|  | $f_{cl}$ |  | 1.01 |
|  | $D$ | mm | 15.00 |
|  | $d$ | mm | 5.00 |
|  | $l$ | mm | 240.00 |
|  | $n$ |  | 9 |
|  | $T_{i}$ | K | 308 |
|  | $T_{r}$ | K | 295 |
|  | $T_{a}$ | K | 295 |

1. Orthogonal factors and levels

| **Level** | ***R_pi_*** | ***R_t_*** | ***T_in_*** | $\boldsymbol{\nu}$ |
| --- | --- | --- | --- | --- |
|  | **m^2^·K/W** | **m^2^·K/W** | **℃** | **L/min** |
| 1 | 0.002 | 0.00 | 5 | 0.4 |
| 2 | 0.003 | 0.03 | 10 | 0.8 |
| 3 | 0.005 | 0.05 | 15 | 1.2 |
| 4 | 0.006 | 0.07 | 20 | 1.6 |

Notes: $R_{t}=0.00$ means no thermal insulation fabric is used on the upper surface of the FHTP.

1. L_16_ (4^4^) orthogonal designed table

| **No.** | $\boldsymbol{R}_{\boldsymbol{pi}}$ | $\boldsymbol{R}_{\boldsymbol{t}}$ | $\boldsymbol{T}_{\boldsymbol{in}}$ | $\boldsymbol{\nu}$ | $\boldsymbol{Q}_{\boldsymbol{s}}$ | $\boldsymbol{\eta}$ |
| --- | --- | --- | --- | --- | --- | --- |
|  | **m^2^·K/W** | **m^2^·K/W** | **℃** | **L/min** | **W** | **%** |
| 1 | 0.002 | 0.00 | 5 | 0.4 | 16.07 | 73.09 |
| 2 | 0.002 | 0.03 | 10 | 0.8 | 21.83 | 86.60 |
| 3 | 0.002 | 0.05 | 15 | 1.2 | 16.79 | 90.57 |
| 4 | 0.002 | 0.07 | 20 | 1.6 | 12.64 | 96.62 |
| 5 | 0.003 | 0.00 | 10 | 1.2 | 20.78 | 83.05 |
| 6 | 0.003 | 0.03 | 5 | 1.6 | 25.63 | 84.09 |
| 7 | 0.003 | 0.05 | 20 | 0.4 | 7.68 | 94.25 |
| 8 | 0.003 | 0.07 | 15 | 0.8 | 16.94 | 91.60 |
| 9 | 0.005 | 0.00 | 15 | 1.6 | 16.14 | 86.69 |
| 10 | 0.005 | 0.03 | 20 | 1.2 | 11.67 | 95.47 |
| 11 | 0.005 | 0.05 | 5 | 0.8 | 24.82 | 85.40 |
| 12 | 0.005 | 0.07 | 10 | 0.4 | 12.83 | 82.84 |
| 13 | 0.006 | 0.00 | 20 | 0.8 | 11.76 | 94.58 |
| 14 | 0.006 | 0.03 | 15 | 0.4 | 10.03 | 80.61 |
| 15 | 0.006 | 0.05 | 10 | 1.6 | 20.00 | 86.87 |
| 16 | 0.006 | 0.07 | 5 | 1.2 | 23.70 | 86.09 |

1. Parameters of TPU laminated fabrics.

|  | **Fabric material** | **Yarn counts** | **Warp/weft yarn density** | **Coating material** | **Thickness** | **Weight** |
| --- | --- | --- | --- | --- | --- | --- |
|  |  | **D** | **threads/inch** |  | **mm** | **g/m^2^** |
| Inner fabric | Nylon | 70 | 127/81 | TPU | 0.15 | 251 |
| Outer fabric | Polyester | 300 | 53/48 |  | 0.35 | 331 |

Notes: Inner fabric contacts skin in practice.

1. The dimensions of the rectangular FHTP.

| $\boldsymbol{n}$ | $\boldsymbol{D}$ | $\boldsymbol{d}$ | $\boldsymbol{n (D+d)}$ | $\boldsymbol{l}$ |
| --- | --- | --- | --- | --- |
|  | **mm** | **mm** | **mm** | **mm** |
| 9 | 15 | 5 | 180 | 242.5 |

1. The stiffness of inner side of the FHTP and lower limb.

|  | **Stiffness (N/m)** | | | | | | |
| --- | --- | --- | --- | --- | --- | --- | --- |
| **No.** | **1** | **2** | **3** | **4** | **5** | **Average** | $\boldsymbol{\sigma}$ |
| **FHTP** | 467.60 | 592.00 | 525.00 | 518.40 | 658.60 | 552.32 | 74.07 |
| **Calf** | 183.20 | 312.00 | 181.40 | 190.20 | 103.40 | 194.04 | 74.87 |
| **Thigh** | 140.00 | 135.00 | 100.00 | 81.00 | 127.00 | 116.60 | 25.19 |

Notes: The stiffness of the FHTP was measured when water was pumped in the FHTP with the flow rate of 0.8 L/min. $\sigma$ represents the standard deviation.

**Supplementary Notes**

**Supplementary Note 1 Deviation of heat transfer model**

As illustrated in **Fig. 2a**, define the infinitesimal length in the direction of fluid flow (channel length) as $dl$, and the change in fluid temperature is $ⅆT_{f}$. The width of fluid channel is $n\left( D+d \right)$. The total heat ($ⅆq_{f}$) taken away by fluid over the infinitesimal length $dl$ is given by:

| $ⅆq_{f}=\frac{ⅆQ_{f}}{n\left( D+d \right)dl}=\frac{c\rho\nuⅆT_{f}}{n\left( D+d \right)dl}$ | (S1) |
| --- | --- |

The heat flux between the fluid and the skin ($ⅆq_{s}$) and between the fluid and the surroundings ($ⅆq_{a}$) can be calculated using **Eq. (S2)** and **Eq. (S3)**, respectively:

| $ⅆq_{s}=\alpha_{s}\cdot\left( T_{i}-T_{f} \right)$ | (S2) |
| --- | --- |
| $ⅆq_{a}=\alpha_{a}\cdot\left( T_{a}-T_{f} \right)$ | (S3) |

From the energy balance equation, we obtain **Eq. (S4)**:

| $ⅆq_{f}= ⅆq_{s}+ⅆq_{a}$ | (S4) |
| --- | --- |

Substituting **Eqs. (S1-S3)** into **Eq. (S4)** and further separating the variables, we get:

| $\frac{ⅆT_{f}}{dl}=\frac{\alpha_{s}\left( T_{i}-T_{f} \right)\cdot n\left( D+ⅆ \right)}{c\rho\nu}+ \frac{\alpha_{a}\left( T_{a}-T_{f} \right)\cdot n\left( D+ⅆ \right)}{c\rho\nu}$ | (S5) |
| --- | --- |

In the heat transfer process within the skin-FHTP-surroundings system, the parameters $n$, $D$, and $d$ are constants for the FHTP. Additionally, when the flow rate of inlet water remains unchanged, the parameters $R_{f}$, $R_{int}$, and $A$ are also constants. Consequently, according to **Eqs. (3)** and **(4)**, the parameters $\alpha_{s}$ and $\alpha_{a}$ are constants as well. Under these conditions, **Eq. (S5)** can be further expressed as **Eq. (S6)**:

| $\frac{ⅆT_{f}}{dl}= \frac{C_{1}T_{i}+C_{2}T_{a}}{c\rho\nu}-\frac{C_{1}+C_{2}}{c\rho\nu}T_{f}$ | (S6) |
| --- | --- |

where $C_{1}=\alpha_{s}\cdot n\left( D+d \right)$ and $C_{2}=\alpha_{a}\cdot n\left( D+d \right)$ are constants.

By solving **Eq. (S6)**, the fluid temperature $T_{f}$ is a function of the channel length ($l$):

| $T_{f}=(T_{in}- \frac{\alpha_{s}\cdot T_{i}+\alpha_{a}\cdot T_{a}}{\alpha_{s}+\alpha_{a}}){\cdot e}^{-\frac{(\alpha_{s}+\alpha_{a})\cdot n\left( D+d \right)}{c\rho\nu}\cdot l}+\frac{\alpha_{s}\cdot T_{i}+\alpha_{a}\cdot T_{a}}{\alpha_{s}+\alpha_{a}}$ | (S7) |
| --- | --- |

The heat transfer rate between skin and the FHTP ($Q_{s}$) and between the FHTP and surroundings $(Q_{a}$) are obtained by integrating along the channel length:

| $Q_{s}=\int_{0}^{l} \alpha_{s}\cdot n\left( D+d \right)(T_{i}-T_{f})dl$ | (S8) |
| --- | --- |
| $Q_{a}=\int_{0}^{l} \alpha_{a}\cdot n\left( D+d \right)(T_{a}-T_{f})dl$ | (S9) |

Substituting **Eq. (S7)** into **Eqs. (S8)** and (**S9)**, the expressions for $Q_{s}$ and $Q_{a}$ are derived as follows:

| $Q_{s}=\frac{\alpha_{s}\alpha_{a}(T_{i}-T_{a})\cdot n(D+d)}{\alpha_{s}+\alpha_{a}}\cdot l+c\rho\nu\cdot\frac{{\alpha_{s}}^{2}\left( T_{i}-T_{in} \right)+\alpha_{s}\alpha_{a}\left( T_{a}-T_{in} \right)}{\left( \alpha_{s}+\alpha_{a} \right)^{2}}\cdot\left( 1-e^{-\frac{(\alpha_{s}+\alpha_{a})\cdot n\left( D+d \right)}{c\rho\nu}\cdot l} \right)$ | (S10) |
| --- | --- |
| $Q_{a}=\frac{\alpha_{s}\alpha_{a}(T_{a}-T_{i})\cdot n(D+d)}{\alpha_{s}+\alpha_{a}}\cdot l+c\rho\nu\cdot\frac{{\alpha_{a}}^{2}\left( T_{a}-T_{in} \right)+\alpha_{s}\alpha_{a}\left( T_{i}-T_{in} \right)}{\left( \alpha_{s}+\alpha_{a} \right)^{2}}\cdot\left( 1-e^{-\frac{\left( \alpha_{s}+\alpha_{a} \right)\cdot n\left( D+d \right)}{c\rho\nu}\cdot l} \right)$ | (S11) |

Adding **Eqs. (S10)** and **(S11)**, total heat transfer rate of the FHTP ($Q_{f}$) is

| $Q_{f}=c\rho\nu\cdot\frac{\alpha_{s}\cdot\left( T_{i}-T_{in} \right)+\alpha_{a}\cdot\left( T_{a}-T_{in} \right)}{\alpha_{s}+\alpha_{a}}\cdot\left( 1-e^{-\frac{\left( \alpha_{s}+\alpha_{a} \right)\cdot n\left( D+d \right)}{c\rho\nu}\cdot l} \right)$ | (S12) |
| --- | --- |

Furthermore, the heat transfer effectiveness ($\eta$) of the FHTP is expressed by **Eq. (S13)**:

| $\eta=\frac{Q_{s}}{Q_{f}}$ $\cdot$100% | (S13) |
| --- | --- |

**Supplementary Note 2 Justification for neglecting heat transfer between dots and skin**

The rectangular FHTP is analyzed to evaluate the heat transfer between the skin and the FHTP. The dimensions of the FHTP are provided in **Table S10**, with a width of 180 mm and a length of 242 mm. Within this 180 $\times$ 242 mm^2^ area, the FHTP consists of welding dots (dark) and fluidic channels (grey blue), as depicted in **Fig. S7a**. The diameter of each dot is 5 mm, resulting in a total dot area of 2472 mm². Given the total area of the FHTP is 43,560 mm², the dots constitute 5.7 % of the total area (499$/$43,560 $\times$ 100%).

From the cross-sectional view illustrated in **Fig. S7b** and **c**, the contact conditions between the FHTP and skin are categorized into three distinct areas: the contact area between the fluidic channel and skin (blue), the non-contact area between the fluidic channel and skin (red), and the non-contact area between the dots and skin (yellow). Based on the calculated area ratio from **Fig. S7a**, the non-contact area between the dots and skin is only 5.7 %. Consequently, this area might be combined with the non-contact area between the fluidic channel and skin to simplify the heat transfer pathway.

The validity of this assumption is confirmed by examining the heat transfer rate between the dots and skin ($Q_{d}$) and its ratio to the overall heat transfer rate ($Q_{f}$), using the established heat transfer model. There are conductive thermal resistances of air gaps ($R_{a}$) and skin ($R_{s}$) (detailed calculations are provided in the **Supplementary Note 3**). The equivalent thermal resistance network is depicted in **Fig. S7d**. $Q_{d}$ can be calculated as:

| $Q_{d}={(T_{i}-T_{d})}/{\left( R_{a}+R_{s} \right)\times0.18\times0.24\times1.1\%}$ |  |
| --- | --- |

where $T_{d}$ is the temperature of the dots, assumed to be equivalent to the fluidic temperature. Since the actual temperature of the dots is lower than the fluidic temperature, the predicted $Q_{d}$ values are higher than the actual ones. The temperature under skin of 3 mm is regarded as a constant ($T_{i}$=35 ℃), representing the core temperature. $R_{a}$ and $R_{s}$ denote the conductive thermal resistances of the enclosed air gap and skin, respectively, measured in m^2^$\cdot$K/W.

The effect of the contact area ratio ($A$) on $Q_{f}$ and $Q_{d}$ under four different inlet water temperatures is depicted in **Fig. S8a** and **S8b**. The flow rate in the model is set at 0.8 L/min. Under same contact area ratios and inlet water temperatures, $Q_{d}$ is significantly lower than $Q_{f}$. The ratio of $Q_{d}$ to $Q_{f}$ indicates that $Q_{d}$ constitutes no more than 5 % of $Q_{f}$ when the contact area ratio ranges from 1% to 99% at inlet water temperatures of 5, 10, 15, and 20°C (**Fig. S8c**). These findings demonstrate that the heat transfer rate between the dots and skin is negligible, and the corresponding heat transfer pathway can be disregarded.

**Supplementary Note 3 Calculation on the thermal resistance**

**Thermal resistance of conductive heat transfer:**

The thermal resistance per unit area (unit: m^2^·K/W) of inner and outer fabric, as well as skin, is calculated based on the Fourier's law of heat conduction:

| $R=\delta/\lambda$ | (S14) |
| --- | --- |

where $\delta$ represents the thickness of fabric or skin (unit: m), and $\lambda$ represents the thermal conductivity of the fabric or skin (unit: W/(m$\cdot$K)).

The shape of air gap between skin and the FHTP is irregular. Therefore, the air gap thickness $\delta_{a}$ is simplified to the vertical distance between skin and welding dots, which is estimated to be ~ 4 mm. The conductive heat transfer in the air gap is predominant when the air gap thickness is below 5 mm ^[1]^. Consequently, the thermal resistance in the air gap is calculated as follows:

| $R_{a}=\frac{\delta_{a}}{\lambda_{a}}$ | (S15) |
| --- | --- |

where $\lambda_{a}$ is the thermal conductivity of air layer, which is 0.023 W/(m$\cdot$K).

**Interfacial contact thermal resistance (**$\boldsymbol{R}_{\boldsymbol{int}}$**):**

The thermal contact resistance between the skin and the FHTP is estimated using the empirical correlation developed by Prasher and Matayabas (PM) for finger-object contact scenarios ^[2]^. In our case, the FHTP is not homogeneous material, unlike the examples in PM’s work. The estimated thermal conductivity of the FHTP is in the range from 0.04 W/(m·K) (inner fabric) to 0.6 W/(m·K) (flowing water) based on the experimental results. Therefore, a modification factor $f_{int}$ for interfacial thermal conductivity ($\kappa$) is introduced into the PM empirical correlation to account for this variability. The modified PM correlation for estimating the thermal contact resistance $R_{int}$ between skin (material 1) and the FHTP (material 2) is expressed by **Eq. (S16)**:

| $R_{int}=83.8\frac{\varepsilon}{0.5\kappa\cdot f_{int}}{(\frac{G}{P})}^{\frac{1}{4}}$ | (S16) |
| --- | --- |

where $P$ is applied contact pressure, $G$ is the shear modulus of the softer material, $\kappa$ is the harmonic mean of the thermal conductivities of the two materials in contact, given by $\kappa=2{\kappa_{1}\kappa_{2}}/{(\kappa_{1}+\kappa_{2})}$. $\sigma$ represents the interface roughness, $\varepsilon={({\varepsilon_{1}}^{2}+{\varepsilon_{2}}^{2})}^{0.5}$, $\varepsilon_{1}$ and $\varepsilon_{2}$ both are Root Mean Square (RMS) roughness. Skin is regarded as the softer material, as it has a low-frequency shear modulus of only 0.4 KPa. Furthermore, the stiffness of the FHTP, thigh, and calf are compared and listed in **Table S10**, indicating skin is softer than the FHTP.

**Thermal resistance of forced convective heat transfer (**$\boldsymbol{R}_{\boldsymbol{f}}$**):**

The convective heat transfer coefficient in the fluid channel, $h_{f}$, defines the efficiency of heat exchange by convection, which can be calculated by:

| $h_{f}=Nu\frac{\lambda_{f}}{D_{h}}$ | (S17) |
| --- | --- |

where $\lambda_{f}$ is the thermal conductivity of fluid (W/(m·K)), and $D_{h}$ is the characteristic dimension (m), which can be calculated from $D_{h}={4S_{ch}}/p$^[3]^. Here, $S_{ch}$ and $p$ are the cross-sectional area and the perimeter of a channel, respectively. The geometry of the FHTP under an internal air pressure of 8 KPa is obtained via 3D scanning technology. From the cross-sectional view, a fluid channel is elliptical, formed by two circular arcs between two adjacent welding dots (**Fig. S6**).

Nusselt number $Nu$ is often expressed by an empirical formula using the Reynolds number ($Re$) and the Prandtl number ($Pr$) depending on the state of the fluid. The typical empirical formulas for laminar and turbulent flow are listed below:

| $Nu=3.66+\frac{0.068(Re\cdot Pr\cdot\frac{D_{h}}{l})}{1+0.04 {(Re\cdot Pr\cdot\frac{D_{h}}{l})}^{2/3}}$ (laminar flow) | (S18) |
| --- | --- |
| $Nu=0.023\cdot{Re}^{\frac{4}{5}}\cdot{Pr}^{\frac{1}{3}}$ (turbulent flow) | (S19) |

where $l$ is the channel length. The Reynolds number ($Re$) indicates the likelihood of fluid turbulence and can be given by:

| $Re=\frac{\rho\upsilon_{avg}D_{h}}{\mu}$ | (S20) |
| --- | --- |

Where $\rho$, $\upsilon_{avg}$, and $\mu$ represent the density (kg/m^3^), average flow velocity (m/s), and kinematic viscosity of the fluid (Pa·s), respectively. The average flow velocity $\upsilon_{avg}$ is related to inlet water flow rate ($\nu$) by the **Eq. (S21)**.

| $\upsilon_{avg}= \frac{\nu}{n\cdot A_{ch}}$ | (S21) |
| --- | --- |

Prandtl number $Pr$ is defined as follows:

| $Pr=\frac{\mu\cdot c}{\lambda_{f}}$ | (S22) |
| --- | --- |

where $c$ is the specific heat (J/(kg·K)). The values for $\mu$, $\lambda_{f}$ and $\rho$ of liquid water under different temperatures are adopted from the studies by Kestin et al.^[4]^ , Ramires et al.^[5]^ and Tanaka et al.^[6]^, respectively.

The thermal resistance of forced convective heat transfer in the fluid channel is calculated by:

| $R_{f}=\frac{1}{h_{f}}$ | (S23) |
| --- | --- |

**The thermal resistance of natural convective heat transfer (**$\boldsymbol{R}_{\boldsymbol{c}\boldsymbol{onv}}$**):**

The natural convective heat transfer coefficient $h_{conv}$ is influenced by the temperature difference between the mean surface temperature of the clothed body ($T_{cl}$) and the ambient air temperature $T_{a}$. According to the empirical formulas, $h_{c}$ can be expressed as ^[7]^.

| $h_{conv}=1.175({T_{cl}-T_{a})}^{0.351}$ | (S24) |
| --- | --- |

Heat transfer coefficient $h_{conv}$ have been determined for the nude skin surface, while clothing increases the area available for heat transfer between the skin and the air environment. Therefore, the natural convective heat flux between the FHTP and the air environment $q_{conv}$ is calculated by modified Newton’s law of cooling:

| $q_{conv}=f_{cl}h_{conv}(T_{cl}-T_{a})$ | (S25) |
| --- | --- |

where $f_{cl}$ is the clothing area factor, representing the ratio of the surface area of the clothed body to the surface area of the nude body. The factor $f_{cl}$ is related to the intrinsic clothing insulation value ( $I_{cl}$, unit: m^2^$\cdot$K/W) and can be determined by ^[8]^:

| $f_{cl}=$1+1.97 $\cdot I_{cl}$ | (S26) |
| --- | --- |

The thermal resistance of natural convective heat transfer $R_{conv}$ is therefore derived as:

| $R_{conv}=\frac{1}{f_{cl}h_{conv}}$ | (S27) |
| --- | --- |

**The thermal resistance of radiative heat transfer** $\boldsymbol{R}_{\boldsymbol{r}}$:

The radiative heat flux between the FHTP and the surroundings is defined as $q_{r}$:

| $q_{r}= f_{eff}f_{cl}\varepsilon\sigma(T_{cl}^{4}-T_{r}^{4})$ | (S28) |
| --- | --- |

where $f_{eff}$ is effective radiation area factor of clothed body, estimated to be 0.7 for sitting position; $\varepsilon$ is the average body surface emissivity; $\sigma$ is the Stefan-Boltzmann constant, 5.67×10^-8^ W/(m^2^·K^4^); $T_{r}$ is the mean radiant temperature of the environment (K). The fourth-order temperature difference can be replaced by a linear temperature difference via introducing radiative heat transfer coefficient ($h_{r}$) (**Eq. (S29)**) ^[9]^:

| $q_{r}= f_{cl}h_{r}(T_{cl}-T_{r})$ | (S29) |
| --- | --- |

Based on **Eqs. (S28)** and **(S29)**, $h_{r}$ is derived as follow:

| $h_{r}=f_{eff}\varepsilon\sigma\frac{T_{cl}^{4}-T_{r}^{4}}{T_{cl}-T_{r}}$ | (S30) |
| --- | --- |

Explicit solutions for $h_{r}$ are seldom derived for clothed subjects due to the difficulties in measuring $T_{cl}$. Consequently, the value of $h_{r}$= 4.7 W/(m²·K) has been broadly adopted as a reasonable whole-body estimate for general purposes ^[10]^. The thermal resistance of radiative heat transfer $R_{r}$ is derived as:

| $R_{r}=\frac{1}{f_{cl}h_{r}}$ | (S31) |
| --- | --- |

**Supplementary Note 4 The value of correction factor (**$\boldsymbol{f}_{\boldsymbol{int}}$**)**

The experimental $Q_{f}$ values fall within the range of the modeled $Q_{f}$ values when the thermal conductivity of the object ($k_{2}$) in the model varies from 0.04 W/(m$\cdot$K) (representing the inner fabric) to 0.6 W/(m$\cdot$K) (representing flowing water). This indicates that the effective thermal conductivity of the FHTP lies between 0.04 and 0.6 W/(m$\cdot$K). To align the model results with the experimental data, a correction factor, $f_{int}$, is introduced into the interfacial thermal conductivity ($k$) within the PM correlation (**Eq. (S16)**). For this modified model, $k_{2}$ is set at 0.6 W/(m$\cdot$K). The $Er$ values for $Q_{f}$ and $T_{out}$ are calculated under different fint values ($f_{int}$ = 1, 0.5, 0.3, 0.35, 0.375, 0.4) (**Fig. S10**). It is observed that the$Er$value for $Q_{f}$ is minimum and less than 10% across four different flow rates when $f_{int}$ is set to 0.375. Therefore, $f_{int}$ = 0.375 is adopted in the modified heat transfer model to ensure value consistency with the experimental results under various flow rates.

**Supplementary Note 5 Experimental validation under various inlet water temperature (**$\boldsymbol{T}_{\boldsymbol{in}}$**)**

The modified heat transfer model is further validated by comparing with experimental results of $Q_{f}$ and $T_{out}$ under different inlet water temperature ($T_{in}$). According to **Eqs. (1) ~ (4)**, when the flow rate is valued as 0.8 L/min, $Q_{f}$ and $T_{out}$ as the function of $T_{in}$ are

| $Q_{f}$ = 38.01$-$1.24 ${\cdot T}_{in}$ | (S32) |
| --- | --- |
| $T_{out}$ = 0.68$+$0.98 ${\cdot T}_{in}$ | (S33) |

Meanwhile, $Q_{f}$ and $T_{out}$ from the experimental measurement have highly linear dependence with $T_{in}$ ($R^{2}>0.99$, **Fig. S11**), which can be given by:

| $Q_{f}$ = 46.20$-$1.78 ${\cdot T}_{in}$ | (S34) |
| --- | --- |
| $T_{out}$ = 1.85$+$0.91 ${\cdot T}_{in}$ | (S35) |

Thus, as shown in **Fig. 2f**, the modified model is highly reliable, as $Q_{f}$ and $T_{out}$ from the model fit those from the experiment.

**Supplementary Note** **6 Deviation of** $\boldsymbol{Q}_{\boldsymbol{s}}$ **and** $\boldsymbol{\eta}$ **with only four parameters (**$\boldsymbol{R}_{\boldsymbol{pi}}$**,** $\boldsymbol{R}_{\boldsymbol{t}}$**,** $\boldsymbol{T}_{\boldsymbol{in}}$ **and** $\boldsymbol{\nu}$**)**

The analytical model for $Q_{s}$ and $\eta$ deviates from **Eqs. (S10) - (S13)**, while maintaining all other parameters constant as specified in **Tables S6** except $R_{pi}$, $R_{t}$, $T_{in}$ and $\nu$. The final expression of model for $Q_{s}$ and $\eta$ is represented by **Eqs. (S36) - (S39)**.

| $Q_{s}=\frac{0.09\alpha_{s}\cdot\alpha_{a}}{0.18 {(\alpha}_{s}+\alpha_{a})}+\frac{69.6\nu\cdot\alpha_{s}\cdot(0.03T_{in}\cdot\left( \alpha_{s}+\alpha_{a} \right)-1.1\alpha_{s}-0.7\alpha_{a})\cdot(e^{-\frac{0.0006\cdot\left( \alpha_{s}+\alpha_{a} \right)}{\nu}}-1)}{{(0.18\alpha_{s}+0.18\alpha_{a})}^{2}}$ | (S36) |
| --- | --- |
| $\eta=\frac{64 \alpha_{s}}{\alpha_{s}+\alpha_{a}}+\frac{0.7\alpha_{s}\cdot\alpha_{a}}{\nu\cdot(\alpha_{s}\cdot(34-T_{in})+\alpha_{a}\cdot\left( 22-T_{in} \right)\cdot(1-e^{-\frac{0.0006\cdot\left( \alpha_{s}+\alpha_{a} \right)}{\nu}})}$ | (S37) |
| $\alpha_{s}=\frac{1}{0.06+R_{pi}+\frac{0.17 R_{int}}{0.17 A+(1-A)\cdot R_{int}}+R_{f}}$ | (S38) |
| $\alpha_{a}=\frac{1}{R_{f}+0.12+R_{t}}$ | (S39) |

**Supplementary Note 7 The correlation of the effectiveness (**$\boldsymbol{\eta}$**) related to the temperature difference**

Since the same fluidic fabric is used for both hot and cold therapy, and the inlet water flow rates are identical, the total thermal resistance ($R_{1}$, in m^2^$\cdot$K/W) between the skin and fluid remains the same for both therapies. Similarly, the total thermal resistance ($R_{2}$, in m^2^$\cdot$K/W) between the fluid and surroundings is also consistent. The heat transfer rate between the skin and fluid ($q_{s}$) and between the fluid and surroundings ($q_{a}$) as follows:

| $q_{s}={(T_{i}-T_{f})}/{R_{1}}$ | (S40) |
| --- | --- |
| $q_{a}={(T_{a}-T_{f})}/{R_{2}}$ | (S41) |

The effectiveness (η) can be expressed as:

| $\eta={q_{s}}/{(q_{s}+q_{a})}$ | (S42) |
| --- | --- |

Substituting the equations above into the effectiveness formula and separating the variables, we derive:

| $\eta=\frac{1}{1+\frac{R_{1}}{R_{2}}\times\frac{T_{a}-T_{f}}{T_{i}-T_{f}}}$ | (S43) |
| --- | --- |

This shows that η depends on the ratio of the temperature difference between the surroundings and fluid ($T_{a}-T_{f}$) to the temperature difference between the skin and fluid ($T_{i}-T_{f}$). A higher ratio results in lower effectiveness.

In cold mode,

$$\frac{T_{a}-T_{f}}{T_{i}-T_{f}}=\frac{21-11}{35-11}=0.42$$

In hot mode,

$$\frac{T_{a}-T_{f}}{T_{i}-T_{f}}=\frac{21-40}{35-40}=3.8$$

These results align with the experimental findings that the effectiveness in cold mode is higher than in hot mode.

**References**

[1] E. Mert, A. Psikuta, M.-A. Bueno, R. M. Rossi, *Int. J. Biometeorol.* **2017**, *61*, 363.

[2] a) K. Rykaczewski, *Temperature* **2019**, *6* (1), 85; b) R. S. Prasher, J. C. Matayabas, *IEEE Transactions on components and packaging technologies* **2004**, *27* (4), 702.

[3] H.-C. Chiu, J.-H. Jang, H.-W. Yeh, M.-S. Wu, *International Journal of Heat and Mass Transfer* **2011**, *54* (1-3), 34.

[4] J. Kestin, M. Sokolov, W. A. Wakeham, *Journal of Physical and Chemical Reference Data* **1978**, *7* (3), 941.

[5] M. L. V. Ramires, C. A. Nieto de Castro, Y. Nagasaka, A. Nagashima, M. J. Assael, W. A. Wakeham, *Journal of Physical and Chemical Reference Data* **1995**, *24* (3), 1377.

[6] M. G. Tanaka, G ; Davis, R ; Peuto, A ; Bignell, N, *Metrologia* **2001-08**, *Vol.38 (4)*, 301.

[7] Y. Kurazumi, T. Tsuchikawa, J. Ishii, K. Fukagawa, Y. Yamato, N. Matsubara, *Building and Environment* **2008**, *43* (12), 2142.

[8] I. Holmér, H. Nilsson, G. Havenith, K. Parsons, *Annals of Occupational Hygiene* **1999**, *43* (5), 329.

[9] F. P. Incropera, D. P. DeWitt, T. L. Bergman, A. S. Lavine, *Fundamentals of heat and mass transfer*, Wiley New York, **1996**.

[10] R. J. De Dear, Arens, E., Hui, Z., & Oguro, M. , *Int J Biometeorol* **1997**, (40), 141.
